# Ag₃PO₄@ZnO kraft lignin composite for optimized photocatalytic degradation of methylene blue using response surface methodology

**DOI:** 10.1038/s41598-025-05597-7

**Published:** 2025-06-20

**Authors:** Marwa S. Abdelkader, Sherif A. Younis, Esraa M. El-Fawal, Hager R. Ali, Hosny Ibrahim

**Affiliations:** 1 Analysis and Evaluation Department, Egyptian Petroleum Research Institute, Nasr City, 11727 Cairo Egypt; 2 Chemistry Department, Faculty of Science, Cairo University, Cairo, Egypt

**Keywords:** Ag_3_PO_4_@ZnO p-n heterojunction, Kraft lignin, Waste valorization, Response surface methodology, Textile wastewater treatment, Environmental chemistry, Photochemistry

## Abstract

This research explores the use of kraft lignin (KL), derived from pulping black liquor waste, as a supportive medium for Ag_3_PO_4_@ZnO (AZ-NC) p-n heterojunction and design a new cost-effective ternary KL-Ag_3_PO_4_@ZnO nanocomposite (AZKL). The aim is to improve its photocatalytic efficiency in treating textile wastewater while tackling environmental issues such as chemical stability, charge carrier separation, and the production of secondary waste during the photocatalytic process. The response surface methodology (RSM) analysis shows that AZKL is highly effective catalyst for methylene blue (MB: 10 - 25 mg/L) dye mineralization, achieving a rapid decolorization (> 98.2% within 40 min) under visible light at a near-neutral pH (7.48) with maintained high catalytic activity across four consecutive cycles. This outstanding performance is driven by the synergistic interplay of AZKL-based photocatalysis and advanced oxidation process using 0.03% H_2_O_2_ co-catalyst. Gas chromatography-mass spectrometry analysis reveals that MB dye degrades stepwise into intermediates such as N, N-dimethyl-p-phenylenediamine, hydroquinone, and formic acid, ultimately mineralizing completely into CO₂ and H₂O. The dominant reactive oxygen species driven this multi-step process are identified as hydroxyl radicals (•OH) and photogenerated holes (h⁺), with H₂O₂ and superoxide radicals (•O₂⁻) playing secondary roles. The data also highlights the multifunctional role of KL support, which enhances charge carrier separation, captures dye molecules, and prevents Zn^2+^/Ag^+^ ion leaching (less than 0.2 ppm) into the treated water during photocatalysis. This is facilitated by the electron-donating polyphenolic hydroxyl groups on the KL surface, which reduce Ag⁺ to metallic silver and stabilize AZ-NC heterojunction under light irradiation, creating Schottky junctions that improve charge transfer efficiency while reducing secondary contamination risks. A practical case study further illustrates the effectiveness of AZKL in treating real textile effluents, as evidenced by the improved biodegradability of residual organic matter, indicated by changes in chemical/biological oxygen demands (COD/BOD) ratios from 2.62 to 1.47 and inhibition tests against E. coli, meeting wastewater discharge standards. The findings emphasize that the AZKL composite could serve as an effective and adaptable photocatalyst for breaking down organic pollutants and treating intricate wastewater systems.

**Supplementary Information:**

The online version contains supplementary material available at 10.1038/s41598-025-05597-7.

## Introduction

**Fig. 1:**
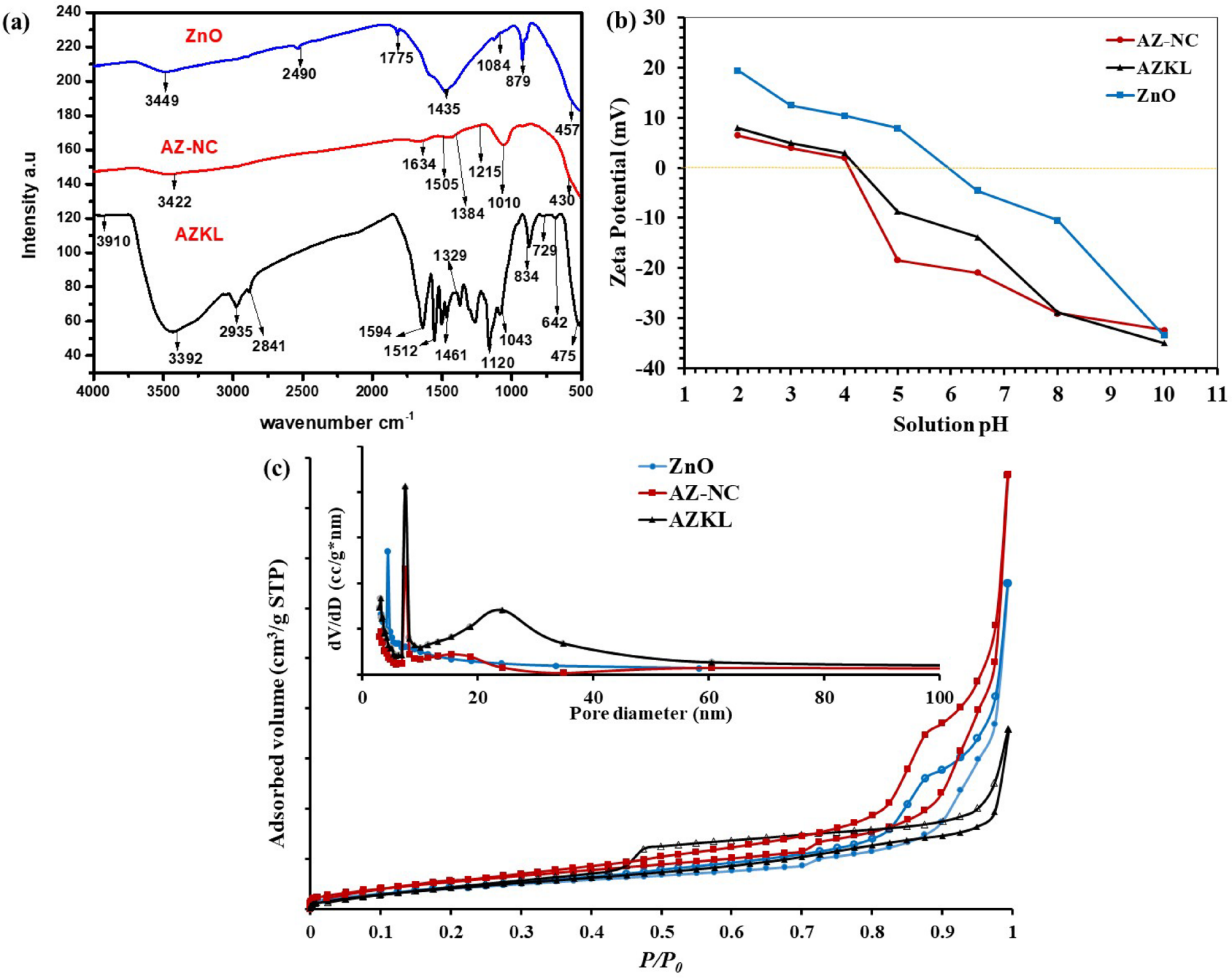
The surface chemistry and textural features of the as-prepared photocatalysts (AZKL, ZnO, and AZ-NC): (**a**) FTIR spectra, (**b**) Zeta potential profiles as a function of solution pH (at 25 ± 1 °C), and (c) N_2_-adsorption-desorption isotherm (inset: pore size distribution curves).

The contamination of water resources by wastewater containing synthetic dyes, particularly from the textile industry, represents a significant environmental challenge. Currently, over 100,000 types of commercial dyes are produced at an annual rate of approximately 700,000 tons. The global consumption of dyes in textile manufacturing reaches about 10,000 tons per year, with an estimated 100 tons of dyes being discharged into wastewater annually ^1^. Many colorful compounds and their degradation intermediates present significant health hazards to humans and other organisms, particularly aquatic life. As a representative synthetic dye model, methylene blue (MB), a cationic dye with molecular formula C_16_H_18_N_3_SCl (Fig. 1S, as illustrated in the electronic supporting information (ESI) file), is widely utilized in various industrial fields, including analytical chemistry, paper, leather, biology (e.g., staining cells), pharmaceuticals, and dyeing process in textile sectors ^2^. Due to its synthetic origin, MB is often present in wastewater, which can lead to health and ecological concerns. Prolonged exposure to MB can cause adverse health effects such as vomiting, nausea, anemia, red blood cell breakdown, serotonin syndrome, tissue necrosis, and ﻿﻿jaundice﻿ (Oladoye et al., 2022). Due to its high toxicity, the permissible limit for MB dye is usually set very low, often at trace levels (less than 0.01 mg/L), depending on local environmental regulations, as there is no standard regulation for MB dye in water ^4,5^. Despite that, the World Health Organization (WHO) and environmental agencies like the United States Environmental Protection Agency (EPA) recommend that the total concentration of synthetic dyes in discharged wastewater should not exceed 1–2 mg/L to avoid aesthetic pollution and ecological damage ^6,7^. Although there is no specific limit for MB, this general guideline for synthetic dyes (< 1–2 mg/L) serves as a reference for evaluating the effectiveness of treatment technologies in cleaning dye-contaminated water. However, MB concentrations in industrial effluents are frequently reported to be between 2 and 50 mg/L, which significantly surpasses the acceptable levels for safe environmental discharge or drinking water standards (Azhar et al., 2022; Mashkoor et al., 2020). Even at levels above trace amounts (higher than 0.5 mg/L), MB is often considered problematic due to its toxicity to aquatic life and its role in water discoloration. Therefore, substantial treatment is necessary to bring MB concentrations down to acceptable levels before wastewater can be released.

Over the last decades, various methods have been explored for the removal of dye compounds in water environments, including advanced oxidation processes ^10^adsorption ^11^electro-coagulation ^12^ozonation ^13^membrane separation ^14^and biological treatment have been studied for degradation of dye compounds ^10,15^. However, these techniques often suffer from drawbacks such as high operational costs due to extensive energy consumption ^10^. Recently, heterogeneous photocatalysis (HPC) has emerged as a sustainable, economical, and efficient approach for treating industrial wastewater. This method leverages semiconductors that absorb solar light to produce a range of oxidizing agents in the aqueous solution, which facilitates the rapid degradation of organic azo dyes into less harmful by-products ^16^. In this context, zinc oxide (ZnO), an n-type semiconductor, is extensively utilized as an effective photocatalyst for degrading various organic pollutants due to its commercial availability, cost-efficiency, and robust thermodynamic stability ^17^. Nevertheless, pure ZnO photocatalysts face significant limitations, including a wide bandgap energy of 3.3 eV that restricts photocatalytic activity to ultraviolet (UV) light, which constitutes only 3–5% of total solar radiation, and a rapid recombination rate of photo-generated electrons and holes (e-/h+) pairs^18^. To address these issues, several ZnO-based junctions with visible-light-active semiconductors have been proposed, such as kraft lignin-Supported ZnO@Ag_3_PO_4 _^19^, Activated carbon onto ZnO ^20^ZnO/GO (graphene oxide) (Puneetha et al., 2021), Fe_3_O_4_/ZnO/GO ^22^ZnO/Bi_2_O_4 _^23^, and KL loaded onto Ag_3_PO_4_@ZnO ^19^ .

Particularly, Ag_3_PO_4_, a p-type semiconductor with a narrow bandgap (E_g_ = 2.36–2.43 eV), high plasmonic response, and quantum efficiency of 90%, has been extensively studied for its ability to enhance photocatalytic properties and redox potential when paired with ZnO in a p-n heterojunction under a broad UV-visible light spectrum ^24^. However, the high photo-corrosion rate and limited stability of Ag_3_PO_4_ nanoparticles (NPs) in aqueous environments significantly restrict the large-scale environmental applications of Ag_3_PO_4_@ZnO p-n heterojunction^24^. Additionally, the use of nanopowder composite photocatalysts in batch wastewater treatment often leads to secondary nano-waste pollutants, as these particles are challenging to recover from treated wastewater and can suffer from aggregation of photogenerated electrons ^26,27^. In recent years, there has been growing research interest in developing Ag_3_PO_4_@ZnO-based ternary systems supported by carbon or polymeric materials. Notable examples include Activated carbon onto ZnO ^20^lignin-based carbon/ZnO composite ^28^and ZnO/lignin-derived flower-like carbon composite (Zhang et al., 2020). The pulp and paper industries currently produce 50–70 million tons of lignin annually, with projections indicating a potential increase to 225 million tons per year by 2030 ^30^. Consequently, as a part of the circular economy and waste utilization in this work, kraft lignin (KL), a widely available by-product extracted from black liquor waste of the pulp and paper industry, was used as a supported material for the Ag_3_PO_4_@ZnO p-n heterojunction (AZ-NC) due to its unique structural and functional properties. As a low-cost and renewable biopolymer, KL offers a sustainable alternative to synthetic carbonaceous materials, aligning with green chemistry principles. The amphiphilic characteristics of lignin also provide both steric and electrostatic stabilization, helping to prevent the agglomeration of AZ-NC nanocatalysts for consistent access to active sites ^31,32^.

In contrast to conventional semiconductor photocatalysts, which often suffer from aggregation and reduced activity over time, this study presents the integration of KL with AZ-NC as a novel approach to address environmental risks linked to nanowaste leaching. The ternary composite of KL-supported Ag_3_PO_4_@ZnO (referred to as AZKL in this work) utilizes KL biopolymer for multiple functions, including acting as a stabilizing agent, electron mediator, and adsorption enhancer, making it a vital component of the composite. For example, the network structure of KL support can encapsulate the Ag_3_PO_4_@ZnO heterojunction, forming a protective barrier that minimizes the release of harmful Ag⁺ ions. This is facilitated by KL’s electron-donating functionalities that help stabilize metallic silver (Ag⁰), thus preserving catalytic activity while preventing secondary pollution. For instance, the presence of numerous oxygen-containing functional groups (such as hydroxyl, carboxyl, and methoxy groups) enhances interactions with photocatalysts, improving charge transfer, minimizing recombination losses, and increasing the generation of reactive species (like •OH and O₂•⁻) necessary for dye degradation. Additionally, KL functions as a photosensitizer by capturing visible light and transferring excited electrons to the AZ-NC semiconductor matrix, which extends the light absorption range and optimizes solar energy utilization. By incorporating KL, a renewable and biodegradable polymer, the AZKL nanocomposite also adheres to green chemistry principles, providing a sustainable solution for water purification. These characteristics highlight the multifunctional role of KL in enhancing the photocatalytic efficiency, stability, and environmental safety of the KL-supported Ag_3_PO_4_@ZnO heterojunction for textile wastewater treatment through synergistic adsorption-photocatalytic pathways in this work. This innovative approach not only utilizes waste-derived materials but also aligns with circular economy principles, offering an efficient and sustainable solution for wastewater treatment.

In this study, a novel ternary KL-supported Ag_3_PO₄@ZnO p-n heterojunction photocatalyst (designated as AZKL) was developed for treating textile dye effluent under visible light, with a focus on achieving high efficiency, environmental safety, and compliance with wastewater discharge standards. The AZKL nanocomposite utilizes the benefits of the Ag_3_PO₄@ZnO p-n heterojunction (termed AZ-NC) along with KL, a biopolymer obtained from black liquor waste, to enhance photocatalytic performance, stability, and water dispersibility, while also promoting eco-friendly waste recycling practices. The study optimized the photocatalytic performance of AZKL compared to the pristine AZ-NC for the visible-light-driven degradation of MB dye (as a representative model dye) using response surface methodology (RSM). This optimization considered the linear and interaction effects of four operational parameters (e.g., pH, catalyst dosage, dye concentration, and H₂O₂ concentration as a co-catalyst) while improving control over process conditions to optimize MB degradation efficiency in scenarios that mimic real-world situations. The research also explored the photocatalytic reaction mechanism by identifying the dominant reactive oxidative species (ROS) in the photocatalytic reaction pathway through scavenger tests, which were further supported by identifying generated intermediates during the degradation process. Unlike traditional binary Ag_3_PO₄@ZnO p-n heterojunction photocatalyst, this ternary AZKL nanocomposite utilizes lignin’s electron-donating properties to stabilize silver nanoparticles, enhance charge separation, and boost ROS generation. This approach enhanced the photocatalytic activities towards the mineralization of textile organic pollutants while also minimizing secondary contamination and ecotoxicity caused by photocorrosion and the dissolution of nanocatalysts during the photocatalytic process, enhancing environmental safety. The effectiveness of this method was confirmed through the evaluation of the long-term stability and practical use of AZKL in treating textile effluent from a nearby industrial site. Overall, this study presents a novel lignin-based photocatalyst that supports sustainable wastewater treatment technologies while addressing the global challenge of industrial dye pollution. The findings contribute to the development of efficient, cost-effective photocatalysts for wastewater remediation.

## Materials and experimental methods

### Materials and characterization of techniques

All the materials in this study were of analytical grade and used as received without further purification. The purity of materials and tools used for the characterization of the prepared photocatalysts is demonstrated in the **ESI** file (***Section S1.1***). Note that MB dye (Fig. 1S **in the ESI file**) has been chosen as the representative pollutant for this study for several reasons. It is frequently present in various industrial wastewater discharges, posing health and environmental hazards. Additionally, MB exhibits high absorption peaks within the visible spectrum (at 670 nm), enabling precise spectrophotometric monitoring of its concentration during photocatalytic treatment processes. As a synthetic dye, it mimics the characteristics of various industrial dyes, such as high water solubility and resistance to degradation, making it an appropriate choice for assessing the effectiveness of photocatalytic systems in wastewater treatment. By focusing on MB dye, this research aims to provide a comprehensive evaluation of the AZKL composite photocatalyst’s performance and its ability to eliminate similar organic pollutants in real-world wastewater treatment scenarios.

### Synthesis and characterization of Ag_3_PO_4_@ZnO/KL nanocomposite

The Ag_3_PO_4_@ZnO/KL nanocomposite photocatalyst (labelled as AZKL) was prepared by a simple ultrasonic-assisted wet-impregnation of Ag_3_PO_4_@ZnO p-n heterojunction (coded as AZ-NC) onto the kraft lignin (KL) support surface, as described in the ESI file (***Section S1.2*)**. All characterization techniques employed to analyze the crystalline structure, texture, morphology, chemical composition, and optoelectronic properties of the synthesized AZKL (in comparison to AZ-NC) are detailed in the ESI file (**Section S1.2).**

### Experimental design and optimization using response surface methodology (RSM)

#### Experimental setup

As a preliminary experimental study to determine the adsorption-photocatalytic synergy, a batch of experimental studies was conducted to evaluate the impact of KL weight ratio on the performance of AZKL nanocomposite towards MB dye removal in dark/light. The adsorption-photocatalytic reaction was performed using a known weight (0.25 g) of the as-prepared catalysts (AZKL (at varying KL ratios), AZ-NC, and ZnO catalysts) suspended in 100 mL of aqueous MB dye solution (10 mg/L MB dye at pH ≈ 6.5 ± 0.5). Prior to the photocatalysis process, the suspended solution matrices were stirred at 100 rpm in the dark for 60 min at room temperature (25 ± 3ºC) to establish adsorption-desorption equilibrium. Following this, they were exposed to a -visible light source (500 W, λ_max_ at 420 nm) for 60 min to initiate heterogeneous photocatalysis (without H_2_O_2_ addition). At regular time intervals (at 5, 10, 20, 40, and 60 min, either in dark or light conditions), 3 mL of the supernatant suspension was collected and centrifuged for analysis using a UV-visible spectrophotometer (model: Jasco V-570) at a wavelength of 670 nm to determine the removal efficiency of MB dye (decolorization efficiency).

For the RSM-based optimization step, the study employed a face-centered composite design (FCCD), a component of RSM, to optimize the photocatalytic performance of the best-performing AZKL (at 1:1 wt% of AZ-NC to KL) in removing MB dye from contaminated water solution (in reference to AZ-NC). The optimization considered the linear, interactive, and quadratic impacts of various operational parameters, including pH (X_1_ = 4–9), initial catalyst dosage (X_2_ = 1.25–5 g/L), H_2_O_2_ concentration (X_3_ = 0–1% (i.e., 0–0.294 mol/L), as a homogeneous co-catalyst), and MB dye concentration (X_4_ = 10–25 mg/L). ***Section S1.3 (ESI)*** explains the rationale for choosing RSM-FCCD and the level of operational variables in this study (e.g., pH and MB dye concentration). Table 1 illustrates the details of 27 RSM-FCCD experimental matrices for photocatalytic evaluation of AZKL and AZ-NC against MB dye removal as a function of interactive effects of operational variable levels. In each RSM-FCCD experimental trial, the mixture was stirred at 100 rpm for 5 min in the dark, followed by visible light irradiation (500 W) for 40 min at a temperature of 25 ± 3ºC. Subsequently, the dye decolorization efficiency was assessed using UV-Vis spectrophotometry. A quadratic model (**Eq.**1) was used to simulate and predict the photocatalytic activities in relation to the effects of operational variables (linear, interactive, and quadratic effects). After eyeach experimental run, the treated wastewater solution (after catalyst decantation by centrifugation) was subjected to UV-visible spectrophotometer analysis for MB dye detection (at _λmax_ = 670 nm).

**Table 1:** Experimental RSM-FCCD matrix for optimizing the ternary AZKL vs. binary AZ-NC photocatalysts against MB dye removal under visible light irradiation.

Run	Experimental variables				Response factor (reaction rate: mg/min)			
					AZ-NC (Eq. 6)		AZKL (Eq. 5)	
	pH (X_1_)	Catalyst mass (X_2_: g/L)	H_2_O_2_ (X_3_: %)	MB dye (X_4_: mg/L)	Actual	Predicted	Actual	Predicted
1	6.5	3.125	1	17.5	0.77	0.77	0.92	0.96
2	4	1.25	0	25	0.79	0.75	0.27	0.30
3	9	5	1	25	0.13	0.15	1.38	1.41
4	4	5	1	10	0.35	0.37	0.54	0.52
5	6.5	3.125	0.5	25	0.50	0.47	1.21	1.14
6	4	1.25	1	10	0.20	0.17	0.48	0.48
7	6.5	5	0.5	17.5	0.88	0.85	1.13	1.19
8	6.5	1.25	0.5	17.5	0.84	0.85	0.76	0.73
9	9	3.125	0.5	17.5	0.88	0.85	0.89	0.89
10	6.5	3.125	0.5	17.5	0.89	0.91	0.99	0.98
11	9	5	0	25	0.11	0.12	1.52	1.51
12	4	1.25	1	25	0.36	0.39	0.37	0.37
13	4	5	0	10	0.42	0.39	0.53	0.51
14	6.5	3.125	0	17.5	0.92	0.89	0.97	0.96
15	4	5	1	25	0.41	0.37	1.19	1.18
16	9	5	1	10	0.51	0.53	0.61	0.56
17	9	1.25	0	10	0.39	0.41	0.54	0.54
18	6.5	3.125	0.5	17.5	0.87	0.91	1.04	0.98
19	4	3.125	0.5	17.5	0.96	0.98	0.668	0.71
20	4	1.25	0	10	0.43	0.45	0.40	0.38
21	9	1.25	1	10	0.27	0.25	0.54	0.55
22	9	1.25	0	25	0.32	0.33	0.60	0.63
23	6.5	3.125	0.5	10	0.50	0.51	0.65	0.75
24	4	5	0	25	0.41	0.46	1.21	1.21
25	9	5	0	10	0.42	0.42	0.63	0.64
26	6.5	3.125	0.5	17.5	0.89	0.91	1.02	0.98
27	9	1.25	1	25	0.11	0.11	0.59	0.61

1 $$\:Y=\:\beta\:0+\:\sum\:_{i=1}^{k}\beta\:i\:Xi+\:\sum\:_{i=1}^{k}\beta\:ii\:X{i}^{2}+\:\sum\:_{i=1}^{k}\sum\:_{j=i+1}^{k}\beta\:ij\:XiXj$$

Where β_0_ is the constant model coefficient, X_i_ and X_j_ are independent variables, β_i_ is the linear coefficient, β_ii_ is the quadratic coefficient, β_ij_ is the interaction coefficient, Y is the predicted efficiency of MB decolorization, and k is the number of independent parameters. Analysis of variance (ANOVA) and determination coefficient (R^2^) were used to evaluate the statistical significance of the model and the quality of fit between experimental data and the simulated model equation based on the outcome of experimental results ^33^. In this study, Design Expert V.10, a free trial, was used to optimize the experimental results, statistical analysis, and model evaluation.

#### Data analysis

Before MB dye solution analysis, the calibration curve of standard MB dye solution was conducted using the UV-visible spectrophotometer at 670 nm to ensure data accuracy and precision. A linear calibration curve was obtained by plotting the absorbance against MB dye concentration (1–30 mg/L, using five calibration points), achieving a correlation coefficient (R²) of ≈ 0.997 with a method detection limit (MDL) of less than 0.5 mg/L. This demonstrates strong linearity and reliability for accurately measuring MB dye concentrations in photocatalytic experiments. All experimental runs were carried out in duplicate to ensure the reproducibility of outcome results with relative standard errors (RSE%) of less than 6.3% at 90% confidence. The photocatalytic activity of the AZKL (relative to pristine AZ-NC photocatalyst) for MB dye removal was evaluated based on multiple metrics such as removal efficiency (RE%: **Eq.** 2), adsorption capacity (Q (mg/g): **Eq.** 3), and kinetic degradation rate (R_mass_ (mg/min): **Eq.** 4).

2 $${\rm RE}\:\%=\:\:\:\:({C}_{i}\:-{C}_{t}\:)/{C}_{i}\times\:100$$

3 $${\rm Q=(C_{i}C_{t})\times\:V/m}$$

4 $$(\:-\frac{dC}{dt}\infty\:\left[C\right];\:-\frac{dC}{dt}={k}_{app}\left[C\right]);{\rm R_{mass}=k_{app}\times\:V}$$

where C_i_ and C_t_ refer to the initial and residual MB concentrations (mg/L) before and after photocatalytic treatment, respectively. k_app_ (min^−1^) is the apparent decay rate constant calculated by pseudo-first-order rate formula, dc and dt represent the change in MB dye concentration (mg/L) and reaction time (min), respectively. V is the reaction volume (L), while m (g) is the catalyst dosage during the experimental study.

### Application case study and stability test

#### Applicability of AZKL for photocatalytic treatment of real textile wastewater sample

Under the optimal conditions established by the RSM-FCCD experimental design, the effectiveness of the AZKL nanocomposite was examined for the photocatalytic treatment of organic dyes in actual textile wastewater sourced from a local industry in Cairo, Egypt, using direct sunlight as an irradiation source. The physicochemical properties of the collected wastewater (e.g., TDS, pH, salinity, density, and biological oxygen demand (BOD_5_, mg/L)) were determined before and after treatment in accordance with standard methods for the examination of water and wastewater ^34^. In this process, using the ideal operating conditions determined by the RSM model analysis, AZKL nanocomposite was added to a 100 mL sample of textile wastewater. The mixture was stirred at 100 rpm in the dark for 5 min, then exposed to sunlight for 120 min on a sunny day (Peak sunlight hours at 12:00–14:00 PM), under ambient temperatures ranging from 28 to 35 °C. The effectiveness of AZKL in treating textile organic pollutants through photodegradation was evaluated using the total organic carbon (TOC), chemical oxygen demand (COD), and biochemical oxygen demand (BOD₅) indicators, according to the standard method for the examination of water and wastewater (APHA, 24th edition). BOD₅ assesses the oxygen used by microorganisms for breaking down organic material over five days, whereas COD and TOC measure the total organic carbon pollutants (both biodegradable and persistent organic carbons). A notable decrease in COD, BOD₅, and TOC levels after treatment signifies the successful elimination of organic pollutants into simpler, less harmful intermediates or fully mineralized into CO₂ and H₂O, showcasing enhanced water quality and the efficiency of the treatment method used.

#### Reusability test

A reusability study of AZKL for treating MB dye solutions (C_0_ = 10 and 25 mg/L) was performed over four consecutive cycles under optimal conditions obtained from RSM-FCCD modeling analysis. After each experimental run, the used AZKL catalyst was collected and washed multiple times with deionized water and 10% ethanol to eliminate any residual organic materials on its surface. It was then filtered and dried at 60 °C for 6 h before being reused in the following experimental cycle.

### ROS radicals identification

Radical scavenger experiments were conducted to identify the ROS involved in the photodegradation of MB dye by AZKL nanocomposite photocatalyst when exposed to visible light. Various scavengers were used, including isopropanol and ethylene glycol (10% v/v) for photogenerated holes (h^+^), sodium carbonate (0.2 M Na_2_CO_3_) for hydrogen peroxide (H_2_O_2_), acetonitrile (100% v/v) for hydroxyl (^•^OH) radical, silver nitrate (0.2 M) for superoxide (•O_2_^−^) radicals, and ascorbic acid (0.2 M) for singlet oxygenO_2_), as outlined in literature ^35^.

## Results and discussion

### Characterization data

**Fig. 2:**
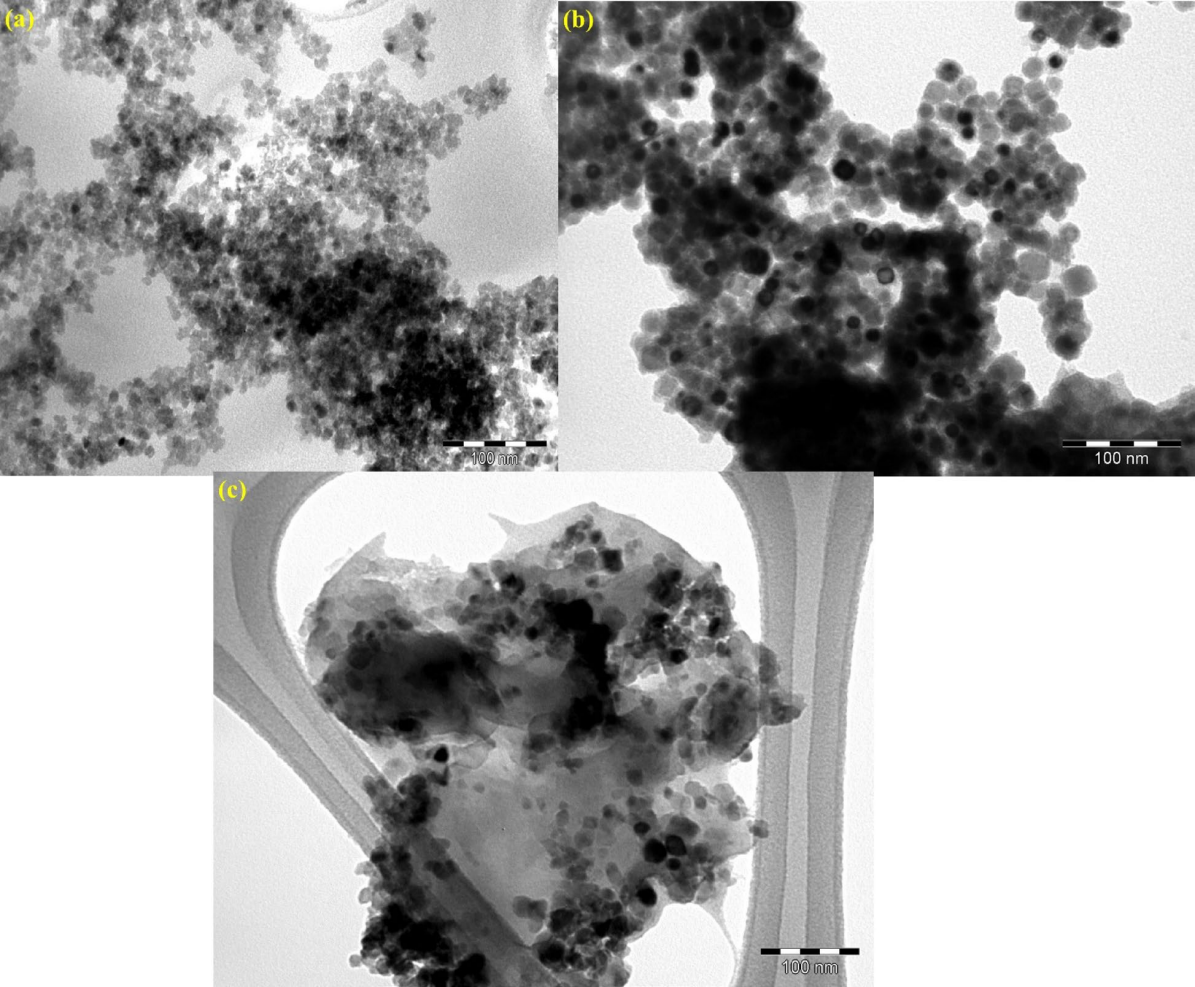
HRTEM images of the as-prepared photocatalysts: (**a**) ZnO NPs, (**b**) AZ-NC, and (**c**) AZKL nanocomposite.

The XRD pattern of the synthesized AZKL nanocomposite (relative to bare ZnO NPs and AZ-NC) is illustrated in Fig. 2S (**ESI**). As can be seen in Fig. 2S, the XRD pattern for pure ZnO displayed clear and sharp diffraction peaks at 2θ angles of 31.92° (100), 34.65° (002), 36.21° (101), 47.85° (102), 56.9° (110), 63.21° (103), and 68.2° (112). These peaks correspond to the hexagonal wurtzite structure of ZnO (JCPDS card No. 36–1451), revealing the high level of ZnO crystallinity with no impurities. In the binary AZ-NC nanocomposite, new small diffraction peaks were observed at 2θ of 20.98° (110), 29.97° (200), 33.53° (210), and 52.25° (310), which correspond to the presence of Ag_3_PO_4_ layer (JCPDS No. 06–0505). This observation is linked with a minor decrease in the intensity of the ZnO diffraction peaks with no significant change in the peak position (compared to pure ZnO), implying that Ag_3_PO_4_ has partially coated the surface of ZnO. Such observation indicates the successful synthesis of the AZ-NC core-shell nanocomposite in this work. When hybridizing AZ-NC with KL support, the XRD pattern of the resultant AZKL ternary nanocomposite showcased similar diffraction peaks to those of AZ-NC. Only a slight reduction in the diffraction peak intensities is recorded due to the presence of amorphous KL support, which partially obscured the crystalline features of ZnO and Ag_3_PO_4_ crystals in the AZKL nanocomposite structure. These observations indicate the successful formation of AZKL nanocomposite without destructive defects in the crystalline structure of ZnO and Agnd ₄ nanocatalysts ^36,37^. The XRD patterns were further analyzed using the Debye–Scherrer’s formula to determine the changes in crystallite size of ZnO (using (101) peak at 2θ = 36.21°) and Ag_3_PO_4_ (using (200) peak at 2θ = 29.97°) crystals after the formation of AZ-NC and AZKL nanocomposites ^38^. The crystallite size of pure ZnO NPs was found to be 53.21 nm, which decreased to 51.35 nm in the AZ-NC nanocomposite and further to 39.7 nm in the AZKL nanocomposite. Additionally, the crystallite size of Ag_3_PO_4_ was estimated at 45.2 nm and 43.8 nm for the AZ-NC and AZKL composites, respectively. The decrease in the size of ZnO crystallites indicates the potential lattice strain after hybridizing with Ag_3_PO_4_ NPs in the binary AZ-NC nanocomposite. Additionally, the inclusion of KL support in AZKL served as a stabilizing agent for both Ag_3_PO_4_ and ZnO nanocrystals, enabling a uniform distribution of nanoparticles (i.e., reducing agglomeration) and preventing large crystal growth during the synthesis process.

The influence of KL-support on the surface chemistry and textural characteristics of AZKL, compared to AZ-NC and ZnO, was examined through various methods such as FTIR spectra, Zeta potential measurements, and N_2_ adsorption-desorption analysis. **In** Fig. 1**(a)**, all the characteristics FTIR bands of Ag_3_PO_4_ and ZnO NPs are observed in the AZ-NC catalyst (e.g., stretching vibrations of P-O-P/O = P-O^−^ groups at 430/1010 cm^−1^ and Zn–O bond at 457/879 cm^−1^). This observation confirms the successful in-situ coating of the ZnO surface with a layer of Ag_3_PO_4_ NPs via the ion exchange method ^39^. The characteristic FTIR peaks of KL were also observed in AZKL nanocomposite at 1120 cm^−1^ (Symmetric S = O (sulfonate) stretching), 1329 cm^−1^ (C-C, C-O, and C = O stretching), 1461 cm^−1^ (the methoxyl C–H bending of aromatic skeleton), 1512 cm^−1^ (the presence of aromatic skeleton), and 2935/2841 cm^−1^ (C-H stretching of aliphatic (methyl, methylene, and methoxyl) groups) (Malhotra et al., 2018; Shi et al., 2019). Additionally, compared with bare ZnO and AZ-NC photocatalysts, the FTIR spectrum of AZKL shows a slight chemical shift in the characteristic absorption bands of ZnO and Ag_3_PO_4_ NPs, ascribing to the effective interaction between AZ-NC and surface oxygenated groups of KL support at their interfaces ^42^.

The Zeta potential measurements illustrated in Fig. 1**(b)** also indicate a strong relationship between the surface charge of all photocatalysts and the pH of the solution. For bare ZnO NPs, the point of zero charge (PZC) is typically around pH 6.2. Below this pH, ZnO surfaces become protonated, leading to positive zeta potentials (ranging from + 7.9 to + 19.5 mV at pH 5 − 2), while deprotonation above the PZC results in negative potentials (from − 10.5 to −33.5 mV at pH 8–10). This pH-dependent charge affects aggregation tendencies, with reduced stability near the PZC due to diminished electrostatic repulsion. The creation of a heterojunction between ZnO and Ag_3_PO_4_ significantly alters surface charge behavior, resulting in a PZC shift for the AZ-NC p-n junction to pH 4.3, which slightly changes to pH 4.5 upon adding KL support to form the ternary AZKL nanocomposite. The inclusion of KL increased the zeta potential to −20.9 mV for AZKL at approximately neutral pH 6.5, compared to −13.8 mV for AZ-NC and − 4.5 mV for pure ZnO. The negative surface charge of AZKL is linked to the carboxyl and phenolic groups in the lignin framework, which enhances electrostatic repulsion and colloidal stability. These results highlight the crucial role of KL support in preventing AZ-NC particle aggregation and ensuring dispersibility in water solution during photocatalysis (Nasseh et al., 2020).

The Textural features of AZKL (relative to bare ZnO and AZ-NC heterojunction) were also examined using N_2_-Adsorption-desorption isotherms in Fig. 1**(c).** Notably, all photocatalysts demonstrated Type IV isotherm behavior along with an H3-H4 hysteresis loop, characteristic of mesoporous materials (pore size ranging from 2 to 50 nm; inset Fig. 1**(c)).** Specifically, the AZ-NC heterojunction exhibited a greater specific surface area (S_BET_ of 62.34 m²/g) compared to ZnO (S_BET_ of 50.9 m²/g), although its total pore volume is slightly less (V_t_ of 0.289 cm³/g) than that of ZnO (V_t_ of 0.301 cm³/g). In contrast, the ternary AZKL nanocomposite demonstrated a significant decline in textural properties (compared to AZ-NC), recording an S_BET_ of 44.27 m²/g and V_t_ of 0.265 cm³/g. The average pore diameter of ZnO, AZ-NC, and AZKL was 7.41, 4.42, and 23.9 nm, respectively. The increased surface area of the AZ-NC heterojunction (relative to pure ZnO) is due to the deposition of smaller, polyhedral Ag_3_PO_4_ NPs on the ZnO surface, resulting in a rougher and more irregular structure. Additionally, the creation of a heterojunction between ZnO and Ag_3_PO_4_ leads to structural configurations like core-shell or aggregates, further enhancing the surface area compared to the smoother surface of pure ZnO ^43,44^. However, the deposition of Ag_3_PO_4_ on the ZnO surface is often associated with partial occupation or obstruction of the pores, which decreases the AZ-NC pore volume (compared to bare ZnO NPs). Moreover, the compact arrangement of Ag_3_PO_4_ on the ZnO surface restricts access to the internal pores, leading to an even further reduction in the total pore volume and average pore size of AZ-NC compared with pure ZnO ^45,46^. As a bulky and amorphous biopolymer, KL also tends to cover the surface of the AZ-NC heterojunction in the ternary AZKL nanocomposite, which obscures some active sites and further decreases the specific surface area and pore volume. However, from a functional perspective, although the decrease in surface area might seem like a disadvantage, KL compensates for this by shifting the pore size distribution towards larger openings and increasing the number of adsorption sites due to its functional groups, such as hydroxyl and methoxy groups ^19,46^. These functional groups enhance the binding of dye pollutants through π-π stacking and hydrogen bonding. The larger pore sizes also facilitate the diffusion of pollutants into the AZ-NC heterojunction catalysts, providing better access to active photocatalytic sites. Together, these factors enhance the efficiency of the ternary AZKL nanocomposite for removing MB dye via both dual adsorption and photocatalytic processes when exposed to visible light (as illustrated in Sect. 3.2).

**Fig. 3:**
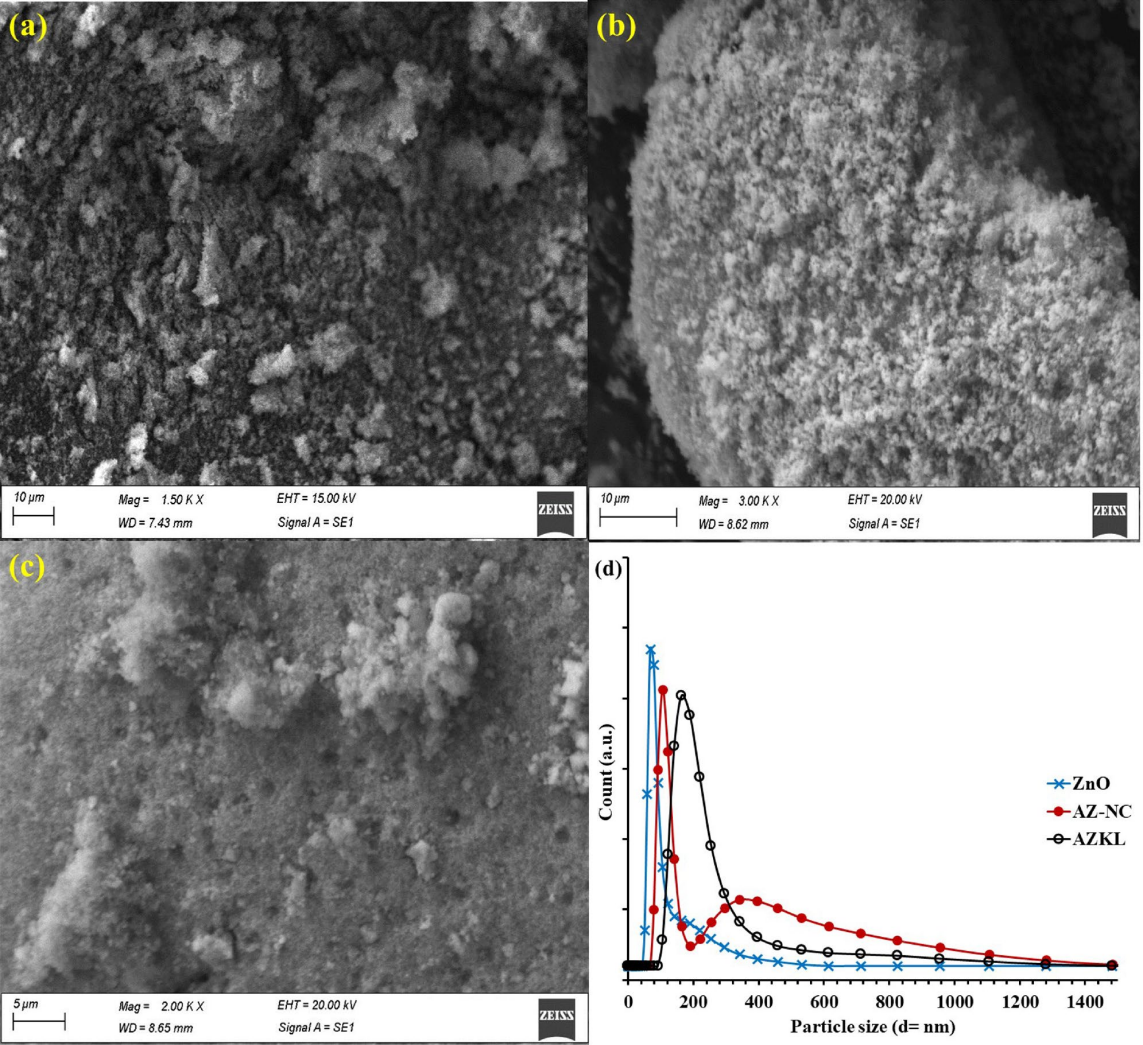
Morphological features of AZKL (compared with ZnO and AZ-NC): (**a**-**c**) SEM micrographs [(**a**) ZnO, (**b**) AZ-NC, and (**c**) AZKL] and (**d**) DLS particle size distribution profiles.

The optical properties of the AZKL and AZ-NC nanocomposites (relative to bare ZnO NPs) were assessed using UV-Vis DRS spectroscopy, as illustrated, as shown in Fig. 3S**(a) (ESI)**. Based on the UV-Vis DRS spectra, the Kubelka–Munk function [(F(R∞)hv)*n* vs. photon energy  (hv, eV)^5^] formula was applied to determine the bandgap energy (E_g_, eV) of the ternary AZKL nanocomposite (in reference to AZ-NC and pure ZnO photocatalysts), as shown in Fig. 3S**(b-c)**. Compared with the bare ZnO photocatalyst, both AZ-NC and AZKL nanocomposites showcased an improvement in the optical response in the whole UV-visible light range (200–490 nm). As predicted, both AZ-NC and AZKL nanocomposites exhibited improved optical responses throughout the entire UV-visible light spectrum (200–475 nm) when compared to pure ZnO photocatalyst, which exhibited a cut-off absorption edge (A_cutoff_) at a round 380–390 nm assigned to its wide bandgap energy (E_g_ of 3.1–3.2 eV) with near-band-edge excitonic transitions (Fig. 3S**(a))**. Remarkably, the A_cutoff_ was extended to 420 nm for the AZ-NC p-n heterojunction. The improved optical response of AZ-NC (compared to ZnO) is mainly ascribed to the plasmonic resonance effects of Ag_3_PO_4_ NPs, which significantly broadens the absorption range into the visible spectrum ^47^. This is supported by the UV-vis DRS and the Tauc plot of pure Ag_3_PO_4_ (Fig. 3S **(b))**, which indicated the capability of synthesized Ag_3_PO_4_ to absorb visible energy with a cutoff edge (A_cutoff_) at 527 nm and a bandgap energy (E_g_) of 2.34 eV. The presence of KL support in the ternary AZKL nanocomposite further expanded the absorption spectrum and resulted in greater absorption intensity throughout the visible range, recording the A_cutoff_ value of 475 nm. There is also a noticeable absorption tail in the 450–750 nm range of AZKL, which suggests that there is interfacial electron coupling occurring between the π-π* transitions in the KL support and the AZ-NC heterojunction ^48^. In Fig. 3S**(c)**, the E_g_ value significantly decreased from 3.22 eV for ZnO to 2.94 eV for AZ-NC p-n heterojunction and then further reduced to 2.72 eV with the addition of KL support (i.e., for the ternary AZKL nanocomposite). The improved optical response of AZKL (relative to AZ-NC and bare ZnO) is attributed to lignin’s photosensitizing effect, which allows it to absorb visible light and transfer excited electrons to the AZ-NC heterojunction, enhancing the overall light-harvesting efficiency. Such an observation reflects the key role of KL’s support on the improved optoelectronic features of AZ-NC, which promote effective charge transfer (i.e., through ligand-to-metal charge transfer (LMCT) interactions) and better light utilization ^19,49^.

**Fig. 4:**
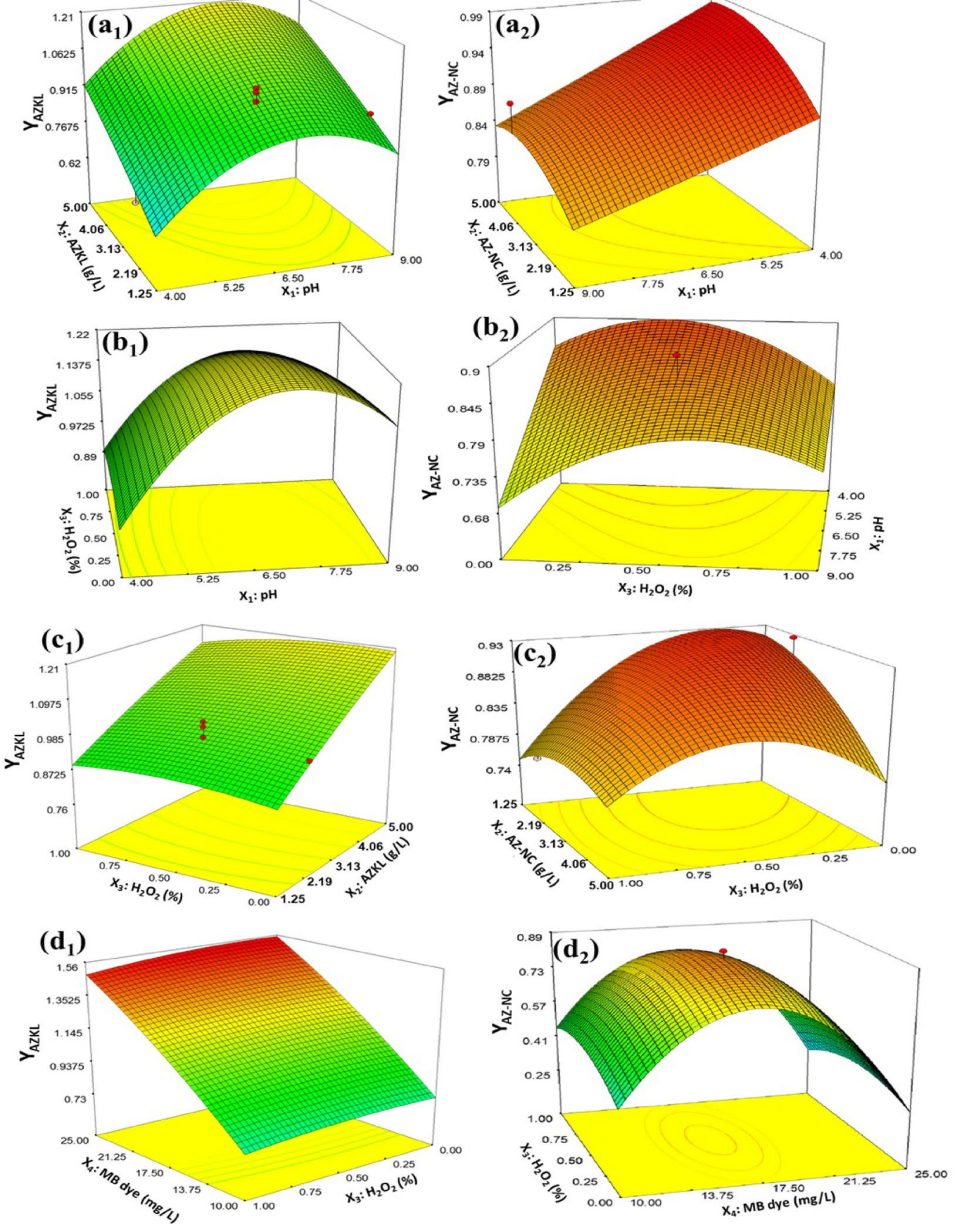
Response surface contour plots of MB dye removal rate by AZKL (a_1_, b_1_, c_1_, and d_1_) and AZ-NC (a_2_, b_2_, c_2_, and d_2_) as a function of 2-way interactive effects of each two operational variables (while keeping the other factors at their mid-level settings): (**a**)X_1_ × _2_, (**b**) X_1_ × _3_, (**c**) X_2_ × _3,_ and (**d**) X_3_ × _4_.

The enhanced optical characteristics of the ternary AZKL nanocomposite (relative to AZ-NC and ZnO catalysts) are validated through photoluminescence (PL) emission analysis, as shown in Fig. 3S**(d) (ESI)**. Pure ZnO exhibited a stronger PL emission intensity with a single broad peak in the 370–520 nm range, which is associated with rapid electron-hole ((e^−^/h^+^) recombination. However, upon the addition of Ag_3_PO_4_, the PL emission intensity of the AZ-NC nanocomposite significantly decreased, indicating that the recombination process is inhibited due to effective charge separation at the p-n heterojunction interface ^50,51^. The TRPL spectra depicted in Fig. 4S**(a)** (**ESI**) indicate that AZKL nanocomposites exhibit a notably longer charge carrier lifetime than both the binary AZ-NC p-n heterojunction and pure ZnO photocatalysts. The average charge carrier lifetimes are 5.26 ns for ZnO, 8.01 ns for AZ-NC, and 9.18 ns for AZKL. The TPC dentistry shown in Fig. 4S**(b)** also confirms that the AZKL nanocomposite exhibited the highest photocurrent density (at ~ 17 µA/cm²), which is 1.61 and 2.33 times higher than that of AZ-NC p-n heterojunction (~ 10.6 µA/cm²) and pure ZnO (~ 7.3 µA/cm²), respectively. The significant improvement in the optoelectronic characteristics of the AZKL nanocomposite, in comparison to the binary AZ-NC heterojunction and pure ZnO photocatalysts, is probably due to the KL substrate’s synergistic effect. The inclusion of KL support in the ternary AZKL nanocomposite introduces additional interfacial interactions, which further lowers emission intensity and suppresses the recombination of photogenerated charge carriers ^52^. In this context, KL, a polyphenolic lignin polymer, likely acts as an electron donor (e.g., –OH and –COOH), capturing holes or reactive oxygen species, thus further minimizing the recombination of photogenerated charge carriers in AZKL (relative to AZ-NC heterojunction)^48,53^. This indicates that the KL biopolymer substrate in the AZKL nanocomposite significantly boosts electron mobility within the AZ-NC heterojunction, enhancing charge transport efficiency and photon absorption when exposed to light. Accordingly, the combined effects of heterojunction-driven charge separation and lignin-mediated scavenging result in a highly effective system for degrading textile pollutants, demonstrating the superior performance of the ternary AZKL nanocomposite compared to pure ZnO and AZ-NC catalysts against MB dye solution (as discussed in Sect. 3.2).

HRTEM analysis was acquired to confirm the interfaces between Ag_3_PO_4_ and ZnO within the AZ-NC heterojunction and its hybrid composite with the KL substrate, as shown in Fig. 2. The HRTEM images indicate that the AZ-NC heterojunction typically features agglomerated polymorphs resembling spherical nanoparticles, which create a close interface between Ag_3_PO_4_ and ZnO, thus improving charge transfer and separation dynamics (Fig. 2). The integration of AZ-NC heterojunction into the KL support allows the carbon scaffold structure of the KL substrate to facilitate an even distribution of AZ-NC nanocomposite within the polymeric matrix of KL, characterized by an amorphous hierarchical structure. This arrangement fosters a strong connection between the KL and AZ-NC heterojunction components, resulting in enhanced photocatalytic efficiency through improved charge separation, an increase in active sites, and potentially better light absorption across a wider UV-visible spectrum. The morphology and particle size distribution of the synthesized photocatalysts were analyzed using SEM and DLS, as illustrated in Fig. 3**(a-d)**. The SEM image in Fig. 3**(a)** reveals that the ZnO clusters have an irregular, rice grain-like shape. The formation of the AZ-NC heterojunction leads to significant aggregation of irregular particles, a result of the morphological changes during the in-situ hybridization of the Ag_3_PO_4_ layer onto the ZnO NPs (Fig. 3**(b)**). In the ternary AZKL nanocomposite, the distribution of the AZ-NC NPs was significantly improved within the sponge-like KL polymeric support matrix, thereby reducing the agglomeration of catalytic sites (Fig. 3**(c)**). DLS profiles presented in Fig. 3**(d**) reveal that ZnO exhibits a particle size distribution ranging from 91.3 to 342 nm, whereas the AZ-NC heterojunction displays a bimodal size distribution between 43 and 106 nm and 122.5–712 nm. When KL support is included (e.g., AZKL), the size distribution expands to 122.4–1281.3 nm due to the microscale, with a higher frequency of smaller particles. The increase in particle size was due to the addition of the Ag_3_PO_4_ layer onto the ZnO surface and the creation of heterostructures with KL biopolymer support, which contributed to the larger overall size of the AZKL nanocomposite particles ^54,55^.

### Photocatalytic MB dye removal by AZKL nanocomposite

#### Effect of KL loading ratio in AZKL on the adsorption-photocatalytic performance

**Fig. 5:**
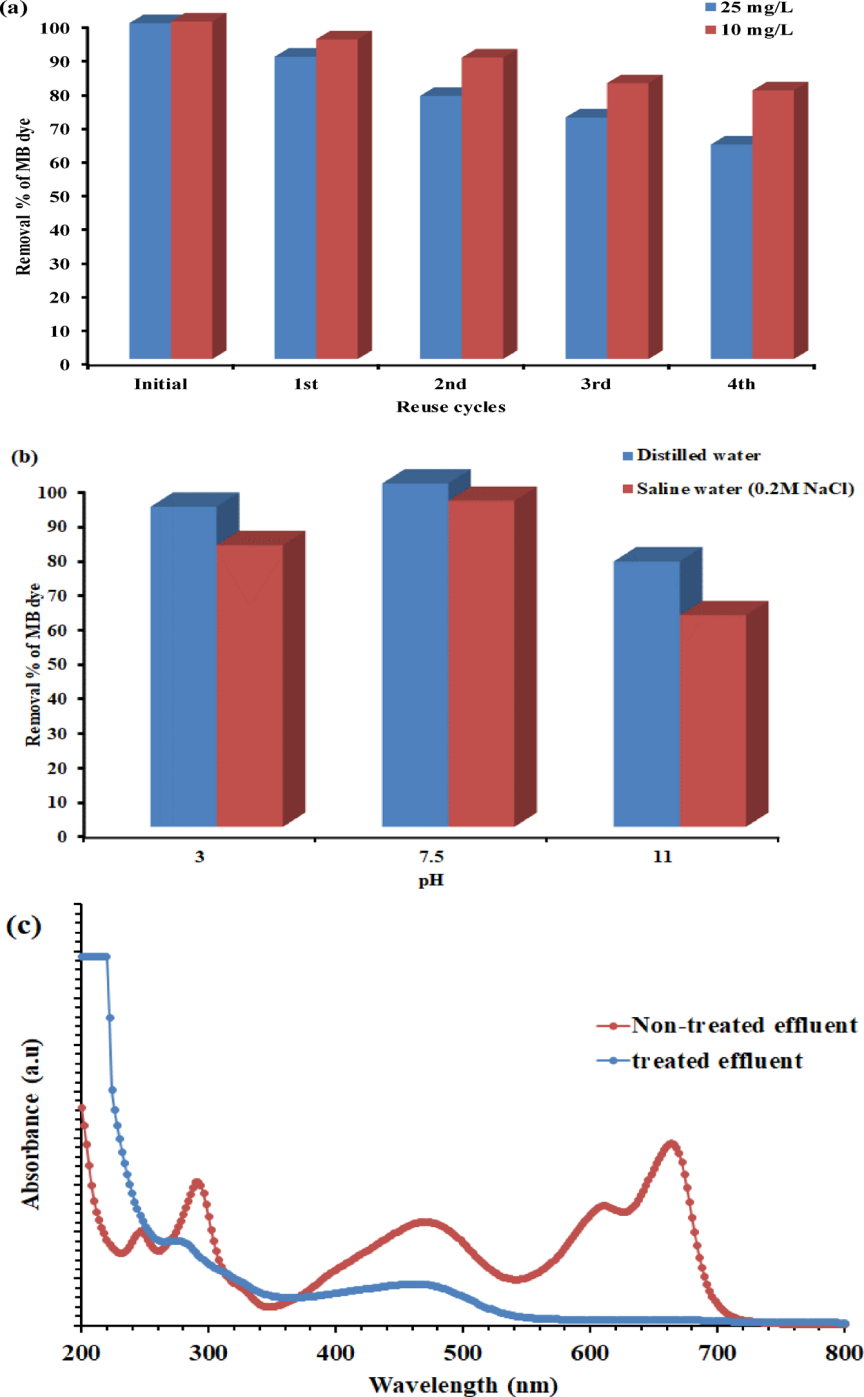
The photocatalytic stability and applicability of AZKL nanocomposite in wastewater treatment: (**a**) reusability cycle against 10 and 25 mg/L MB dye removal across 4 reuse cycles under optimum conditions (pH 7.48, 0.03% H₂O₂, and 4.92 g/L of catalyst), (**b**) performance stability in extreme pH and saline conditions against 25 mg/L MB dye (at 0.03% H₂O₂ and 4.92 g/L of catalyst), and (**C**) the UV-Vis spectrum for actual wastewater before and after solar-light induced photocatalytic treatment.

It is well known that KL support significantly impacts the adsorption and photocatalytic response of AZ-NC heterojunction against MB dye removal, where the molecular structure of MB dye plays a pivotal role in its interaction with the components of AZKL nanocomposite. MB is a cationic dye characterized by a planar aromatic structure, which facilitates strong π-π stacking interactions with aromatic components of KL support in the AZKL photocatalyst ^56,57^. Additionally, the positive charge on the MB molecule enhances electrostatic attraction to negatively charged oxygen-containing surface functional groups on KL, promoting effective adsorption onto the AZKL surface ^57^. This adsorption is a critical precursor to efficient photocatalytic degradation, as it brings the dye molecules into close proximity with reactive species generated by the catalyst under light irradiation. The efficiency of this process is influenced by the specific functional groups present in both the dye and the catalyst material. Therefore, before the RSM optimization study, this study systematically examined the effect of KL loading range in the AZKL nanocomposite (at 1: 0.2, 1: 0.5, and 1: 1 wt% of AZ-NC: KL) on the adsorption and photocatalytic removal efficiency of MB dye (relative to pristine AZ-NC and ZnO catalysts) under both dark and light conditions, as shown in Fig. 5S **(ESI**). This preliminary study aims to achieve the highest photocatalytic performance while maintaining the structural integrity of the developed AZKL nanocomposite photocatalysts for the enhanced treatment of dye-contaminated wastewater.

Figure 5S (**ESI**) illustrates that the adsorption and photocatalytic degradation of MB dye improved as the KL loading ratio in AZKL increased from 1: 0.2 to 1: 1 wt%. All AZKL nanocomposites demonstrated better performance in removing MB dye compared to both AZ-NC and ZnO photocatalysts due to the combined effects of adsorption and photocatalysis. Specifically, in the first 5–10 min of dark conditions, the AZKL (1: 1 wt% ratio) exhibited the fastest adsorption removal of MB dye (36.8–41%) compared to that of AZ-NC (3.6–6.9%) and ZnO (1.47–4.6%) photocatalysts. Notably, the adsorption of MB onto the AZKL(1: 1) nanocomposite showed no notable change over time in dark conditions. However, the efficiency of adsorption decreased by 1.14 to 1.6 times and by 3.3 to 4.8 times when the KL loading ratio in the AZKL structure was reduced from 1: 1 to 1: 0.5 and 1: 0.2 wt%, respectively (Fig. 5S). This verified the key role of KL surface functional groups on the enhanced electrostatic interactions of cationic MB dye molecules. Under visible light-induced photocatalysis, as projected, the AZKL(1: 1) demonstrated the highest photocatalytic degradation efficiency for MB dye, achieving 84.8% dye removal after 60 min of light irradiation (compared to 34.8 and 67.40% MB degradation efficiency by ZnO and AZ-NC photocatalysts, respectively). Lowering the KL content below an optimum ratio of 1: 1 wt% in AZKL resulted in a notable decline in the photocatalytic effectiveness by around 5 to 12.5% (Fig. 5S), emphasizing the essential function of KL in altering surface chemistry and optoelectronic characteristics. From Fig. 5S (**ESI**), the overall adsorption-photocatalytic performance of the prepared catalysts for MB dye removal can be ranked as AZKL(1: 1) > AZKL (1: 0.5) > AZK(1: 0.2) > AZ-NC > ZnO.

To verify that the photocatalytic activity observed in the AZKL(1:1) nanocomposite was not affected by impurities in the black liquor waste-derived KL support, a control experiment was conducted using commercial alkali-lignin (97% pure, Sigma-Aldrich) under the same experimental conditions. The findings indicated that both the commercial and waste-derived KL supports significantly enhanced the adsorption and photocatalytic performance of the AZKL(1:1) nanocomposite for the removal of MB dye, with minimal differences in their effectiveness under light OFF/ON conditions (Fig. 5S, **ESI**). Indeed, the application of the as-synthesized waste-derived KL support resulted in a modest enhancement in the adsorption and photocatalytic performance of AZKL(1:1) for removing MB dye, achieving an improvement of approximately 1.45–8.3% compared to using commercial alkali lignin (Fig. 5S, **ESI**). This observation indicates the slight contribution of impurities present in KL-derived from pulp liquor (e.g., leftover sulfur compounds and inorganic salts) on the adsorption and photocatalytic removal of MB dye by AZKL(1: 1) compared to pure commercial alkali lignin support.

The adsorption behavior of MB dye onto all photocatalysts is ascribed to the promoted electrostatic attraction between negatively charged photocatalytic surfaces (PZC < solution pH 6.5 ± 0.5) with the positively charged MB molecules. The addition of KL further enhances the adsorption behavior of AZKL by providing more adsorption sites and optimizing charge distribution between KL and AZ-NC nanocatalysts in dark/light conditions ^58^. At optimum AZ-NC: KL ratio of 1: 1 wt%, the AZKL(1: 1) nanocomposite exhibited a synergistic effect between Ag_3_PO_4_@ZnO and KL, utilizing the π-π stacking interactions and hydrogen bonding from KL functional groups (e.g., hydroxyl and methoxy groups) to improve cationic MB dye adsorption in the dark (compared to bare AZ-NC and ZnO). Upon light irradiation, the higher KL content at the 1:1 also enhanced charge separation and reactive oxygen species generation, accelerating the photodegradation of MB dye onto AZKL (1: 1) nanocomposite. Accordingly, it is suggested that the improved photocatalytic efficiency of AZKL(1: 1) (in reference to AZ-NC and ZnO) is due to the efficient charge separation enabled by the AZ-NC heterojunction and the production of reactive oxygen species, such as ∙OH radicals, which is further improved by the electron-donating characteristics of KL support. The impurities present in KL derived from pulp liquor can also add surface diversity and functional groups, which alter surface chemistry and charge dynamics to facilitate MB dye removal efficiency slightly ^19,59^. These findings underscore the role of the nanocomposite structure in treating MB dye-contaminated wastewater solution through a synergistic adsorption-photocatalysis mechanism.

## ***Analysis of RSM-FCCD for optimizing MB dye photocatalytic degradation using tertiary AZKL versus binary AZ-NC photocatalysts***.

### Design of experiment and model validation

This research utilized the RSM-based FCCD design, consisting of 27 experimental runs, to investigate and optimize the influence of four independent variables [pH (X_1_), catalyst dose (X_2_), H_2_O_2_ concentration (X_3_), and dye concentration (X_4_)] on the photocatalytic removal rate of MB dye (mg/min) using AZKL nanocomposite (at a 1:1 weight ratio of AZ-NC to KL) compared to AZ-NC heterojunction. Table 1 presents the RSM-FCCD design matrix for the 27 experimental runs conducted to optimize AZKL (compared to AZ-NC) for the photocatalytic degradation of MB dye in relation to the interactive effects of four operational variables (X_1_ – X_4_) under visible light exposure. The experimental findings presented in Table 1 indicated that the degradation rate of MB on AZKL and AZ-NC can be accurately predicted using a second-order polynomial model (**Eq.** 1) with a high regression coefficient (R^2^) value of higher than 0.98. In contrast, the linear and two-factor interaction (2FI) models failed to reflect the complexity of the photocatalytic degradation under tested operational conditions, yielding lower R^2^ values of < 0.21 and inadequate predictive accuracy. Accordingly, two second-order polynomial models were developed to mathematically simulate how the experimental operational variables (X_1_ –X_4_) influence the photocatalytic degradation rate of MB dye (mg/min) onto AZKL (Y_−AZKL_; **Eq.** 5) and AZ-NC (Y_−AZNC_; **Eq.** 6) photocatalysts under visible light irradiation, as follows:

5 $$\begin{aligned} & {Y}_{AZKL}=1.310+\:\sum\:_{i=1}^{4}\left(\:0.0837{X}_{1}+0.314{X}_{2}\:0.0399{X}_{3}\:+\:0.5155{X}_{4}\right)\\ &\quad+\:\sum\:_{i=1}^{4}(-0.188{X}_{1}^{2}-0.089{X}_{2}^{2}-0.0216\:{X}_{3}^{2}-0.041{X}_{4}^{2})+\:\sum\:_{i=1}^{4}\sum\:_{j=i+1}^{4}(-0.0098\:{X}_{1}{X}_{2}\\ &\quad-0.0219{X}_{1}{X}_{3}+0.0434{X}_{1}{X}_{4}-0.0451{X}_{2}{X}_{3}+0.391{X}_{2}{X}_{4}-0.007{X}_{3}{X}_{4})\end{aligned}$$

6 $$\begin{aligned} &\:{Y}_{AZNC}=0.7317+\:\sum\:_{i=1}^{4}\left(-0039-0.428{X}_{2}+0.0469{X}_{3}-\:0.117{X}_{4}\right)+\:\sum\:_{i=1}^{4}(0.001\\ &\quad-0.254{X}_{2}^{2}-0.0733\:{X}_{3}^{2}-0.42{X}_{4}^{2})+\:\sum\:_{i=1}^{4}\sum\:_{j=i+1}^{4}(0.0336{X}_{1}{X}_{2}+0.0317{X}_{1}{X}_{3}-0.0935{X}_{1}{X}_{4}\\ &\quad+0.1294{X}_{2}{X}_{3}-0.1167{X}_{2}{X}_{4}-0.0187{X}_{3}{X}_{4})\end{aligned}$$

(Eq. 6)

Where X_1_, X_2_, X_3_, and X_4_ represent solution pH, catalyst dose (AZKL or AZ-NC, g/L), H_2_O_2_ molar concentration, and MB dye concentration (mg/L), respectively. The constant values and their signs in the regression models above indicate how operational variables influence photocatalytic performance positively (positive sign) or negatively (negative sign). As a fact, these mathematical models successfully serve as a predictive tool for optimizing photocatalytic systems in relation to the linear ($\:\sum\:_{i=1}^{k}\beta\:i\:Xi$), and quadratic ($\:\sum\:_{i=1}^{k}\beta\:ii\:X{i}^{2})$, and interactive ($\:\sum\:_{i=1}^{k}\sum\:_{j=i+1}^{k}\beta\:ij\:XiXj$) effects of the operational parameters (X1-X4) on the photocatalytic degradation rate of MB using AZKL and AZ-NC nanocomposite photocatalysts. Within these models (**Eqs.** 5 and 6), the linear terms ($\:\sum\:_{i=1}^{k}\beta\:i\:Xi$) effectively illustrate the direct effects of each operational factor on the MB dye degradation rate, whereas the quadratic terms ($\:\sum\:_{i=1}^{k}\beta\:ii\:X{i}^{2})$ account for the non-linear relationships that emerge as these parameters interact with one another. Additionally, the interaction terms (($\:\sum\:_{i=1}^{k}\sum\:_{j=i+1}^{k}\beta\:ij\:XiXj$) reveal the synergistic or antagonistic relationships between different variables, which are essential for optimizing the photocatalytic performance of the designed AZKL and AZ-NC against MB dye removal.

The statistical significance and reliability of these terms were validated using analysis of variance (ANOVA), ensuring that the model can be reliably applied to predict outcomes under similar experimental conditions. The ANOVA analysis presented in Table 2 shows that the regression analysis for the photocatalytic activity of the AZKL nanocomposite (Y_AZKL_, **Eq.** 5) produced a high R² value of 0.988, which is closely aligned with the adjusted R² (0.975) and predicted R² (0.95), indicating its strong predictive capability. Similarly, the mathematical model predicting the photocatalytic activity of the binary AZ-NC heterojunction photocatalyst (Y_AZNC_, **Eq.**6) achieved a high R² value of 0.992, with adjusted and predicted R² values of 0.981 and 0.942, respectively, confirming the model’s reliability. The ANOVA analysis presented in Table 2 also shows that the statistical model F-values are 71.29 for Y_AZKL_ (**Eqs.** 5) and 98.11 for Y_AZNC_ (**Eq.** 6), both with p-values under 0.0001. The difference between the Predicted R² and Adjusted R² values is minimal, remaining under 2%, indicating the accuracy and the reliability of the experiment model’s design. The coefficient of variation (C.V.) is noted at 7.13% for the AZ-NC and 6.65% for the AZKL photocatalyst, reflecting deviation errors below 10% and underscoring the high adequacy of the developed model in predicting experimental findings. Additionally, adequate precision levels were recorded at 30.311 for AZ-NC and 30.754 for AZKL, both above the acceptable threshold of 4.00, which further emphasizes the model’s robustness ^60^. The F-values from the analysis indicate that the initial concentration of the MB dye has the most significant effect on color removal, overshadowing the impact of the other parameters examined in the study. The higher model F-test values (compared to the critical F-test value of 2.82) suggest that both models are robust and appropriate for accurately predicting the response factor, with only a 0.01% chance that such high “Model F-Values” could arise from random variation in MB degradation rates, given the operational variables considered within the defined experimental parameters (Table 2). Terms with a “Probability > F” (p-value) below 0.05 are deemed significant at a 95% confidence level in this work. In this case, both models demonstrated high adequacy in capturing the intricate interactions between the operational variable and the photocatalytic response of AZ-NC and AZKL nanocomposite photocatalysts for MB dye removal, as evidenced by the lack-of-fit (LOF) test results, which were not significant (*p* = 0.054 to 0.179; Table 2).

**Table 2:** ANOVA statistics for the derived polynomial models that simulate the photocatalytic removal of MB dye onto AZKL (Eq. 5) and AZ-NC (Eq. 6) photocatalysts.

Source model	df	sum of squares (SS)		mean square (MS)				F-test value				*P*-value		
		AZ-NC	AZKL	AZ-NC	AZKL		AZ-NC		AZKL		AZ-NC		AZKL	
model	14	2.0496	2.7919	0.1464	0.1994		98.11		71.293		˂0.0001		˂0.0001	
X_1_-pH	1	0.0065	0.0305	0.0065	0.0305		4.38		10.915		0.0582		0.0063	
X_2_-dose	1	0.0105	0.0056	0.0105	0.0056		7.02		2.0152		0.0212		0.1812	
X_3_-H_2_O_2_	1	0.0096	0.0069	0.0096	0.0069		6.42		2.4782		0.0262		0.1414	
X_4_-dye conc.	1	0.06	1.1594	0.06	1.1594		40.19		414.48		˂0.0001		˂0.0001	
X_1_ × _2_	1	0.0045	0.0003	0.0045	0.0004		3.02		0.1372		0.1076		0.7175	
X_1_ × _3_	1	0.016	0.0076	0.016	0.0077		10.74		2.7436		0.0066		0.1235	
X_1_ × _4_	1	0.1397	0.0301	0.1397	0.0301		93.65		10.756		˂0.0001		0.0066	
X_2_ × _3_	1	0.0669	0.0081	0.0669	0.0081		44.86		2.9083		˂0.0001		0.1139	
X_2_ × _4_	1	0.0545	0.6116	0.0545	0.6116		36.49		218.64		˂0.0001		˂0.0001	
X_3_ × _4_	1	0.0056	0.0008	0.0056	0.0008		3.76		0.3124		0.0763		0.5865	
X_1_^^2^	1	0	0.0907	0	0.0907		0.002		32.425		0.9688		0.0001	
X_2_^^2^	1	0.0104	0.0013	0.0104	0.0013		6.96		0.4528		0.0216		0.5137	
X_3_^^2^	1	0.0138	0.0012	0.0138	0.0012		9.27		0.4301		0.0102		0.5243	
X_4_^^2^	1	0.4537	0.0043	0.4537	0.0043		304.03		1.5516		˂0.0001		0.2367	
Residual	12	0.0179	0.0336	0.0015	0.0028									
Lack of fit	10	0.0177	0.0322	0.0018	0.0032		17.99605		4.9533		0.0538		0.1796	
Pure Error	2	0.0002	0.0013	0.0001	0.0006									
Cor Total	26	2.0676	2.8255											
Statistical model analysis:														
Metrics	AZ-NC	AZKL	Metrics	AZ-NC	AZKL									
Std. Dev.	0.0386	0.053	R^2^	0.991	0.988									
Mean	0.541	0.805	Adj R^2^	0.981	0.974									
C.V. %	7.135	6.567	Pred R^2^	0.942	0.947									
PRESS	0.12	0.150	AdeqPrecision	30.311	30.754									

**Fig. 6:**
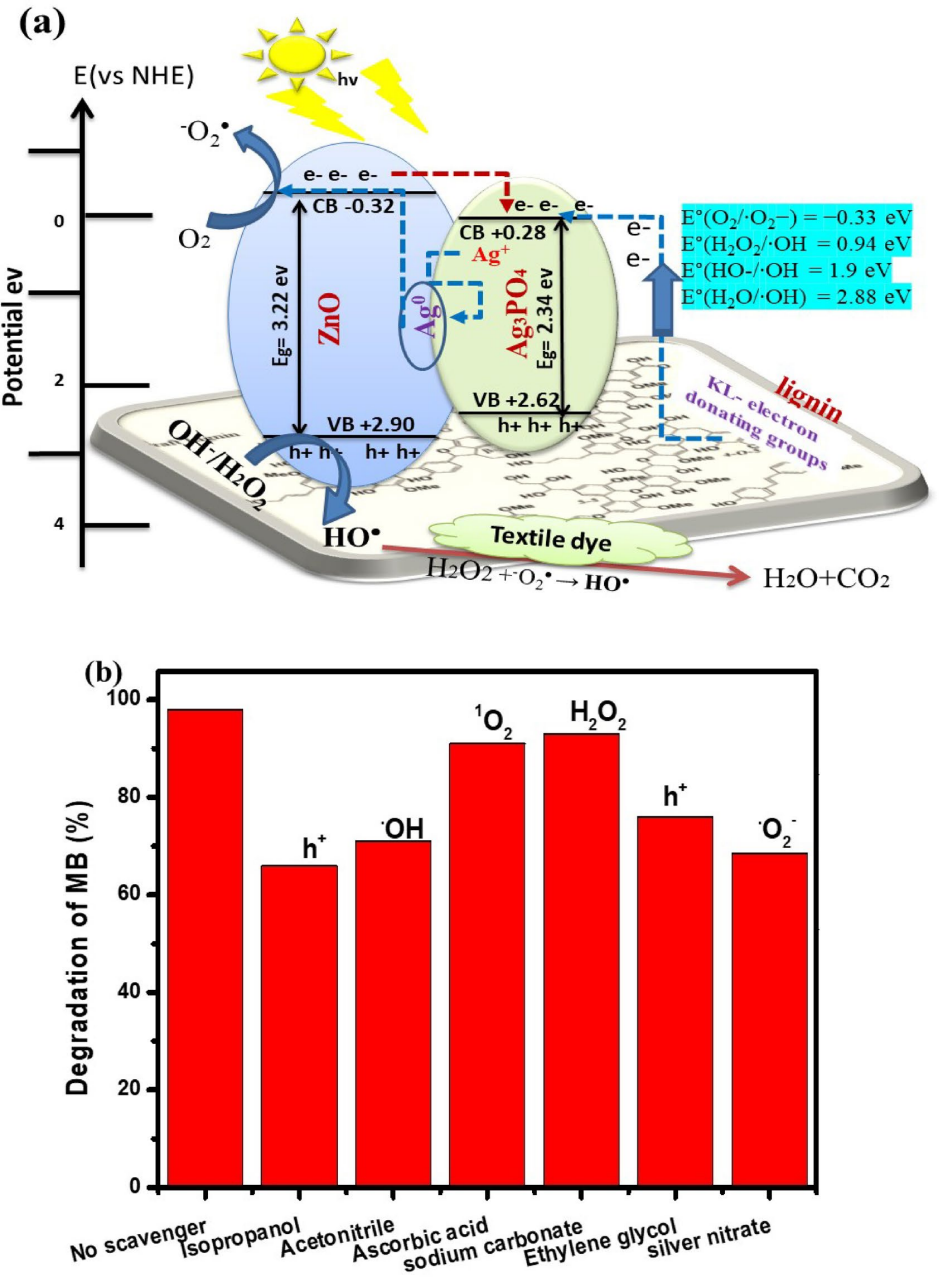
Photocatalytic reaction mechanism of MB dye onto AZKL under light irradiation: (**a**) charge transfer mechanism and (**b**) the radical scavenging test.

To further validate the model’s adequacy, Fig. 6S (**ESI**) shows the normalized residual, experimental versus predicted response trend, Box-Cox, and normal probability plots for the developed models. The normal probability plots (Fig. 6S (a), **ESI)** of the residuals displayed a clear linear trend, supporting the assumption of normality in the dataset. Additionally, an examination of the residuals against the predicted values revealed no discernible patterns (Fig. 6S (b-c), **ESI**), indicating that the data follows the homoscedasticity principle, with residual variations remaining consistent at around ± 3 of the predicted values. Additionally, in the evaluation of the Box-Cox plots (Fig. 6S (d), **ESI**) , it was determined that the 95% confidence interval surrounding the optimal lambda value—identified as the point of minimum on the transformation curve—includes the value of 1. Consequently, this suggests that no specific transformation is warranted for the models being analyzed, as the data appears to meet the necessary criteria without further adjustment. The comparison between predicted and experimental MB degradation rate values also showed a strong linear correlation, with deviations kept below 3%. This small margin of error not only reinforces the reliability of the predictions but also confirms the overall robustness of the mathematical model in predicting the photocatalytic performance of AZ-NC and AZKL photocatalysts in the removal of MB dye under visible light irradiation in complex experimental conditions. Collectively, these in-depth analyses reinforce the robustness of the model and affirm its suitability for the dataset being analyzed. Such consistent alignment between theoretical and practical outcomes also underscores the effectiveness of the RSM-FCCD employed in this analysis in simulating the photocatalytic performance of AZ-NC and AZKL for MB degradation under various conditions.

### Analysis of variance (ANOVA): factor effects and interaction analysis

The study utilized a statistical ANOVA approach along with response surface contour plots to analyze the linear and interactive effects of various factors on the photocatalytic degradation rate of MB dye using AZKL (compared to AZ-NC), as shown in Table 2 and Fig. 4. It is important to note that the model term factors, or operational variables, were deemed statistically significant and had a substantial impact on the photocatalytic response (i.e., MB degradation rate) if their F-test value exceeded the critical F-test value of 2.82 (with a p-value less than 0.05) in this study ^61^. Accordingly, to evaluate the linear and interactive effects of tested operational variables on the photocatalytic performance of AZKL compared to AZ-NC for MB degradation, a ranking was established based on the F-test, p-values from Table 2, and the coefficient constant values in the developed model equations (**Eqs.** 5 and 6). Based on the F-values in Table 2, the impact of operational variables on the photocatalytic degradation rate of MB dye is prioritized in the following order when using the ternary AZKL nanocomposite (Y_AZKL_): initial MB dye concentration (X_4_) is the most influential, followed by solution pH (X_1_), H_2_O_2_ concentration (X_3_), and catalyst dosage (X_2_). However, this ranking changes slightly with the binary AZ-NC heterojunction photocatalysts (Y_AZ−NC_), where the order becomes X_4_ > X_2_ > X_3_ > X_1_.

Specifically, the main factors influencing the Y_AZKL_ response are the solution pH and the initial concentration of MB, as indicated by F-test values of 10.91 and 414.48, with p-values of 0.006 and less than 0.0001, respectively (Table 2**)**. In contrast, the effects of catalyst dosage and H_2_O_2_ concentration were deemed statistically insignificant (*p* > 0.05) on the Y_AZKL_ response factor. Comparatively, all four variables significantly influenced the photocatalytic degradation rate of MB dye with AZ-NC (Y_AZ−NC_), showing lower p-values of less than < 0.05 and higher F-test values ranging from 4.38 to 40.19. Among these, the presence of H_2_O_2_ co-catalyst was identified as the most important factor, demonstrating a positive synergistic effect on the Y_AZ−NC_ response factor. Consequently, the photocatalytic degradation rate of MB dye with AZ-NC was notably enhanced due to the combined influence of H_2_O_2_ with pH (X_1_ × _3_) and/or the amount of AZ-NC catalyst (X_2_ × _3_), with p-values ranging from 0.007 to 0.0001 and F-values between 10.74 and 44.85 (Table 2). Whereas, in the case of AZKL, the Y_AZKL_ response factor was notably affected by the interactions between solution pH and initial MB concentration (X_1_ × _4_; F-value of 93.65, *p* < 0.0001), as well as the interaction between the interaction between catalyst dosage and initial MB concentration (X_2_ × _4_; F-value of 36.49, *p* < 0.0001). These interactions were the most impactful on the Y_AZKL_ response factor. In contrast, the combined effects of H_2_O_2_ as a co-catalyst with catalyst dosage showcased a low to non-significant impact on the Y_AZKL_ response, as indicated by p-values between 0.14 and 0.18 and F-test values between 2.015 and 2.47. This suggests that the H_2_O_2_ and catalyst dosage had a minimal influence on the photodegradation rate of MB dye when using AZKL nanocomposite, likely due to the high capability of AZKL for MB dye removal through a synergistic adsorption-photocatalysis process, especially when compared to pristine AZ-NC (Table 2). These ANOVA findings are corroborated by the 3D response surface contour plots presented in Fig. 4.

The 3D response surface contour plots in Fig. 4 demonstrate the two-way interaction between the operational variables (X_1_ - X_4_) and their impact on the photocatalytic degradation rate of MB dye using AZKL and AZ-NC photocatalysts. These plots help in understanding the relationships among the operational variables and in determining the optimal combination of their values to enhance the photocatalytic results. Figure 4**(a)** illustrates the 2-way interactive effect of solution pH and catalyst dosage (X_1_ × _2_) on the MB degradation rate using AZKL (Y_AZKL_) and AZ-NC (Y_AZNC_) photocatalysts. For AZKL, the Y_AZKL_ improves significantly with rising catalyst dosage (up to 5 g/L) and pH levels from acidic (pH < 5) to near neutral (pH 6.5–7.5). Beyond this optimal pH, the Y_AZKL_ significantly declines with either an increase or a decrease in solution pH. In contrast, the AZ-NC photocatalyst performs best at an acidic pH < 5 with a moderate catalyst dosage of 3–4 g/L (Fig. 4**(a))**. A higher amount of AZ-NC (e.g., 5 g/L) also leads to decreased MB degradation rates (Y_AZNC_) at all pH levels, likely due to agglomeration and reduced active sites for ROS generation. In Fig. 4**(b)**, the interaction between pH and H_2_O_2_ (X_1_ × _3_) also plays a crucial role, with pH being the primary factor for optimizing Y_AZKL_ regardless of H_2_O_2_ concentration. As a matter of fact, there is a slight enhancement (under 10%) in the degradation rate of MB by AZKL (Y_AZKL_) at a neutral pH of 6.5–7.5 with an increase in H_2_O_2_ concentration from 0 to 1%, suggesting its minimal impact on the decolorization rate of MB dye when using AZKL nanocomposite. As a fact, the performance of AZKL drops when the pH rises from 6.5 to 9, while varying H_2_O_2_ levels has little effect on its efficacy across pH levels 4 to 9 (Fig. 4**(b)**). Compared with the ternary AZKL nanocomposite, the MB degradation rate on the binary AZ-NC photocatalyst is significantly influenced by the variation between pH and H_2_O_2_, achieving the highest Y_AZNC_ at an acidic pH of around 4 with a moderate H_2_O_2_ concentration of 0.5% but decreasing with increases in both H_2_O_2_ (from 0.5 to 1%) and pH (from 4 to 9) (Fig. 4**(b)**).

At near neutral pH 6.5–7.5, the improved MB dye removal onto AZKL is likely ascribed to the positive synergy between the adsorption and the photocatalytic processes, which is aided by the functionalized KL support. In this case, the negatively charged oxygenated groups onto KL support facilitated favorable electrostatic interactions of positively charged MB molecules from the water solution. These surface functionalities also play a role in stabilizing the AZ-NC heterojunction catalyst and enhancing charge separation, thereby optimizing the redox reaction process. However, when the pH increased to alkaline conditions (pH 8–9), the photocatalytic performance of AZKL decreased due to two possible reasons: (i) potential deprotonation of oxygenated groups on the KL surface (e.g., phenolic and carboxylic sites) that disrupts the charge density and charge carrier separation between KL and AZ-NC ^62,63^ and (ii) the dissociation of H_2_O_2_ into hydroperoxide (HO_2_•^-^) anions, which have a lower oxidative capacity than.OH radical (i.e., reducing MB removal rate at alkaline pH level)^64,65^. This indicates that the electron-donating properties of KL support in the ternary AZKL are more influential than H₂O₂ in generating ROS during photocatalysis, making the latter less significant when utilizing AZKL for treating MB dye contaminated solution at near neutral pH conditions. This is evidenced by the minimal interaction observed between the dosage of AZKL nanocomposite and the concentration of H_2_O_2_, as shown in Fig. 4**(c**). As can be seen in Fig. 4**(c**), the AZKL nanocomposite exhibits robust performance against MB dye with increasing catalyst dosage, achieving maximum degradation rates at higher doses (e.g., 5 g/L), regardless of H_2_O_2_ concentration. However, at the highest catalyst dosage, the Y_AZKL_ response value decreases slightly by 2.7% with elevated H_2_O_2_ (> 0.5%), likely due to agglomeration or light scattering effects caused by excess KL and scavenger effects of high H_2_O_2_ molecular level for the photogenerated •OH radicals ^64,66^.

On the other hand, the higher effectiveness of AZ-NC photocatalyst at an acidic pH of 4–5 can be attributed to the protonation of the AZ-NC catalyst’s surface, which leads to a stronger electrostatic attraction to the positively charged MB dye molecules, thereby speeding up its degradation rate when H_2_O_2_ is added to the reaction system (i.e., a synergy of heterogeneous-homogeneous catalysis). At low pH, H_2_O_2_ is also stable in its molecular state and serves as a potent oxidizing agent, promoting the formation of ·OH radicals via electron transfer reactions onto AZ-NC surface active sites ^67,68^. These reactive ·OH radicals play a major role in the photocatalytic degradation of MB dye. In addition, acidic conditions hinder the recombination of electrons and holes, which promote s^69^ effective charge separation and enhances the oxidation of H_2_O_2_ into •OH radicals ^64,66^during photocatalysis (i.e., accelerating MB degradation rate) (Zúñiga-Benítez et al., 2016). However, it should be noted that an increase in AZ-NC catalyst dosage from 4 to 5 g/L led to a dramatic decline in the removal rate of MB dye (Y_AZNC_) due to the possible particle agglomeration, limiting light penetration and access to active sites (Fig. 4**(c**)). This issue is intensified at low and elevated H_2_O_2_ levels (i.e., zero and 1%; Fig. 4**(c**)). High levels of H_2_O_2_ result in radical scavenging reactions, such as the reaction between H_2_O_2_ and •OH radicals, which produces the less effective HO_2_•^-^ anions, leading to a drop in overall efficiency of AZ-NC for MB dye removal ^64,66^. Conversely, the generation of ROS decreases at low H₂O₂ concentrations, leading to a further decline in performance. As pH increases, the performance of AZ-NC heterostructure photocatalysts drops due to the potential dissociation of H_2_O_2_ into water and oxygen, which limits its availability for generating •OH radicals and reduces its oxidative power. Moreover, higher hydroxyl anions in alkaline conditions can saturate the AZ-NC catalyst surface, hindering photoactivation and charge transfer mechanisms during photocatalysis ^71^. The electrostatic repulsion between the negatively charged photocatalytic surface and OH^−^ anions in an alkaline environment could also hinder the formation of ·OH radicals and reduce the MB degradation rate onto both AZ-NC and AZKL photocatalysts ^72^.

**In** Fig. 4**(d)**, the surface contour plots for the interactive effects of H₂O₂ concentration and initial MB dye concentration also declared the higher efficacy of AZKL(relative to AZ-NC) in treating highly contaminated MB dye solution (up to 25 mg/L), regardless of H₂O₂ levels. As seen, the ternary AZKL nanocomposite exhibits stability against variations in initial MB dye concentration due to the presence of KL, which offers numerous functional groups that enhance its adsorption capacity and prevent saturation, even at high dye levels. In this scenario, the KL support facilitates the pre-concentration of dye molecules on the surface of the AZ-NC catalyst within the ternary ZKL structure, reducing the dependency on ROS generated by H₂O₂. As a result, H₂O₂ has a limited effect on the performance of the ternary AZKL system, even at elevated concentrations (> 0.5%), since the primary mechanisms for dye removal are the electrostatic and π-π interactions between the MB dye molecules and the KL support ^73,74^. In contrast, AZ-NC binary heterostructure exhibits a strong dependence on initial MB dye and H₂O₂ concentration for effective photocatalysis, achieving the maximum Y_AZNC_ at moderate concentration levels of MB dye (15–20 mg/L) and H_2_O_2_ (0.3–0.5%). However, excessive dye concentrations (> 20 mg/L) or extreme H_2_O_2_ levels (> 0.5%) can impair the effectiveness of the AZ-NC system. This is due to the limited adsorption capacity of AZ-NC for MB dyes and the susceptibility of active sites to saturation with adsorbed dye molecules, which hinders efficient dye degradation ^75,76^. Higher concentrations of MB dye can also cause excessive light absorption, known as the inner filter effect, which decreases photocatalytic efficiency by restricting photon penetration and the generation of reactive species onto AZ-NC photocatalysts. Besides, excessive H_2_O_2_ (> 0.5%) leads to radical scavenging effects (e.g., ·OH reacting with H₂O₂ to form less reactive HO₂·^−^), reducing oxidative efficiency ^77^. These outcomes indicate that the ternary AZKL nanocomposite is more versatile, maintaining stable performance across a broader spectrum of pH levels and catalyst amounts. This contrasts with the bare AZ-NC heterostructure photocatalyst, which has restricted optimal conditions and a dependency on H_2_O_2_ that limits its practical application.

### Optimization of experimental design

The optimization of the photocatalytic efficiency of AZKL and AZ-NC photocatalysts for the removal of MB dye was performed using RSM-FCCD and ANOVA analysis through Design Expert software. The goal was to determine the optimal operating levels to achieve the highest MB dye removal rate at near real-world conditions. According to the model predictions (**Eqs.** 5 and 6) with a desirability level greater than 0.9, the optimal conditions for maximizing MB removal using the AZKL photocatalyst were determined to be a catalyst dosage of 4.92 g/L, a pH of 7.48, and an H_2_O_2_ concentration of 0.03%, assuming a higher initial MB dye concentration of 25 mg/L in the effluent. Conversely, due to the adverse effect of initial dye concentration on the performance of AZ-NC, the ideal conditions for enhancing the removal of 20 mg/L MB dye with AZ-NC were found to be a catalyst dosage of 1 g/L, an H_2_O_2_ concentration of 0.18%, and a pH of 5.34. Under these optimal conditions, the projected photocatalytic removal rates of MB dye were 98.6% (1.56 mg/min) for AZKL and 82.4% (1.09 mg/min) for AZ-NC at an agitation speed of 100 rpm under UV-visible light irradiation. To assess the accuracy of the RSM models’ predictions, further experiments were conducted under the suggested optimal conditions for both catalyst systems. Experimentally, the MB dye removal efficiency onto AZKL and AZ-NC photocatalysts was measured at 99.4% and 84.9%, which is in close agreement with the predicted values, resulting in a relative error of less than 3%. These findings validate the robustness and reliability of the RSM-FCCD models in determining optimal conditions and their applicability in real-world scenarios.

The effect of agitation speed on the photocatalytic efficiency of AZKL (relative to AZ-NC) was also thoroughly examined under optimized conditions with different agitation speeds (100, 150, 200, and 250 rpm), as shown in Table 1S **(ESI).** Note that the agitation speed plays a crucial role in controlling the mass transfer, catalyst distribution, and the interaction between the dye and catalyst surface during photocatalysis. In the case of the ternary AZKL nanocomposite, agitation has a limited effect because of the composite’s high adsorption capacity, achieving 97.5–98.2% MB dye removal at 100 to 150 rpm and maintaining over 95% efficiency even at 250 rpm. This stability is attributed to the π-π stacking of KL support, which helps concentrate dye molecules on the AZ-NC catalyst’s surface, thus decreasing the dependence on hydrodynamic mixing for effective mass transfer. Conversely, the pristine AZ-NC p-n heterojunction photocatalyst is found to be highly sensitive to agitation rate, with a maximum MB degradation efficiency of 90.4% at 200 rpm, but this drops sharply to 78.5% at 250 rpm due to particle clumping and decreased contact time at the interface.

Additionally, the performance of the AZKL nanocomposite is compared with commercial benchmark photocatalysts (Degussa TiO_2_ (P25) and ZnO) for MB dye removal at optimum conditions and 100 rpm agitation speed, as detailed in Table 1S. The results revealed that the AZKL nanocomposite outperformed both commercial TiO₂ (P25) and ZnO as benchmarks against MB dye, even after extending the light irradiation for 60 min. Notably, AZKL demonstrated nearly 99.4% removal of MB dye in less than 40 min, outperforming the photocatalytic efficiency of Degussa TiO₂ (achieved 81.3% removal) and commercial ZnO (reached 68.7% removal) after 90 min of light exposure. The k_app_ value of the AZKL nanocomposite for the degradation of MB dye was also measured at 0.113 min⁻¹, showing an improvement of 6 to 9 times compared to TiO_2_-P25 (0.0186 min⁻¹) and ZnO (0.0126 min⁻¹). The effectiveness of AZKL was also assessed for the adsorption and photocatalytic removal of cationic MB dye versus anionic methyl orange (MO) dye under optimized conditions, as shown in Table 2S **(ESI).** The findings reveal notable variations in the efficiency and kinetic removal rate of both dyes, which are influenced by the surface charge characteristics of the AZKL nanocomposite and the electrostatic interactions with the anionic/cationic dyes. As seen in Table 2S (**ESI**), the AZKL nanocomposite displayed significantly better performance in eliminating cationic MB dye than anionic MO dye, both in darkness and under light. In dark conditions, the removal efficiency of MB dye was 54% with a pseudo-first-order kinetic rate (k) of 0.014 min^−1^. Notably, AZKL exhibited a higher kinetic removal rate for MB dye than MO dye (k = 0.006 min^−1^, with RE = 28.7%) by 2.3 fold of magnitude in dark conditions. When switching to light irradiation, the removal efficiency for MB increased by 45.% within 40 min, reaching 99.4% (k_app_ = 0.113 min^−1^), while MO dye removal only achieved 68.7% after 60 min (k_app_ = 0.02 min^−1^), as shown in Table 2S (ESI).

Note that the outstanding performance of the AZKL nanocomposite in this study (compared to Ag_3_PO_4_@ZnO (AZ-NC) p-n heterojunction and benchmark photocatalysts) can be attributed to several combined factors: (1) the polyphenolic structure of the KL support provides multiple adsorption sites through π-π stacking and hydrogen bonding, which aids in concentrating MB molecules at catalytic interfaces; (2) the formation of the AZ-NC heterojunction on the KL support, which contains electron-donating groups, improves charge separation and significantly reduces electron-hole recombination compared to bare ZnO and TiO2; and (3) the optimized bandgap of AZKL (E_g_ 2.72 eV) and the presence of KL as a photosensitizer enable effective harvesting and utilization of UV-visible light energy, leading to an increased generation of charge carriers for enhanced photocatalytic reactions. According to fundamental concepts of surface chemistry and interfacial interactions, the AZKL nanocomposite surface acquires a negative charge at the experimental pH of 7.48, which is above its PZC value of 4.5. This results in a strong electrostatic attraction to the positively charged MB⁺ dye (cationic thiazine dye, Fig. 1S**(a), ESI)** while simultaneously repelling the negatively charged MO⁻ dye. The presence of KL, with its aromatic structure, also enhances this selectivity by allowing π-π stacking interactions with a planar automatic structure of the MB dye, concentrating the cationic MB dye molecules at reactive sites. Comparatively, in Fig. 1S**(b) (ESI)**, the non-planar structure of the MO dye and the presence of electron-withdrawing sulfonate groups limit the strength of its π-π interactions with KL functional groups, which in turn affects the formation of stable intermediates in photocatalysis. This combination of electrostatic attraction and π-π interactions results in a significantly higher adsorption efficiency for MB (54.0% vs. 28.7% in the dark) and improved photocatalytic degradation efficiency (99.4% vs. 68.7% under light). Additionally, the pre-concentration of MB near the photocatalytic sites (AZ-NC heterojunction) leads to a more effective use of the photogenerated charge carriers and ROS radicals to accelerate the rate of MB degradation vs. MO dye (0.113 min⁻¹ vs. 0.022 min⁻¹). These results affirm fundamental principles of interfacial chemistry in hybrid photocatalytic systems and emphasize the significance of altering surface charges to develop selective adsorbent-photocatalyst composites aimed at removing specific pollutants. Additionally, the AZKL nanocomposite produced is an effective substitute for traditional photocatalysts in the treatment of organic pollutants from textiles, making it a promising advanced option for treating industrial wastewater.

### Photocatalytic stability and performance comparison

To investigate the photocatalytic stability of the AZKL nanocomposite photocatalyst, identified as the top performer, its effectiveness in degrading MB dye at concentrations of 10 and 25 mg/L was assessed over four reuse cycles, as detailed in Sect. 2.4.2. This analysis aimed to evaluate its performance under both low and high organic dye pollutant loads, as shown in Fig. 5**(a)**. At low MB concentration (10 mg/L), the composite demonstrated fast degradation kinetics, reaching more than 98.7% removal within just 10 min (k_app_ of 0.208 min^−1^) under optimum conditions (pH 7.48, 0.03% H₂O₂, and 4.92 g/L of catalyst). This high efficiency is attributed to the abundance of active sites available on the AZKL catalyst surface for capturing low dye load via adsorption and photodegradation synergy. At elevated concentrations of 25 mg/L MB dye, the MB degradation efficiency achieved 99.4% after 40 min with a decrease in the reaction rate (k_app_) from 0.208 to 0.113 min^−1^. The decrease in photocatalytic reaction rate is primarily caused by the saturation of the catalyst’s active sites, limiting additional adsorption and photodegradation at high dye loading in the reaction mixture. Additionally, higher dye concentrations resulted in greater turbidity, which diminished light penetration in the solution and subsequently reduced the production of ROS (i.e., a slight decline in photocatalytic rate over time). Despite this, the AZKL nanocomposite maintained good efficiency over the first and second cycles, achieving more than 78% and 89.2% removal for MB dye at 25 and 10 mg/L, respectively. Following the fourth cycle, the effectiveness of MB removal using AZKL declined by approximately 36% and 20.5% for high (25 mg/L) and low (10 mg/L) concentrations of MB dye, respectively, compared to the initial cycle performance. The decrease in performance of the ternary AZKL nanocomposite after four cycles can be attributed to several factors, including interfacial strain, photocorrosion, and limitations in adsorption. At higher concentrations of MB dye, the active sites on the composite become saturated more quickly during the initial cycles, which hampers the complete regeneration of the catalyst surface for later cycles. Additionally, the accumulation of intermediate products, such as aromatic compounds or organic fragments, resulting from the simultaneous photocatalytic degradation of high MB dye concentrations can obstruct active sites or compete with MB molecules for adsorption, thereby diminishing photocatalytic efficiency . The increased turbidity at high MB concentrations also restricts light penetration, which reduces the generation of ROS and further contributes to the decline in performance. In contrast, at lower MB dye concentrations, the decrease in efficiency is less significant since the catalyst surface is not saturated, allowing for more stable adsorption and photodegradation across multiple cycles. Nevertheless, some minor deactivation occurs due to gradual surface fouling from residual intermediates or slight structural changes in the composite, such as the partial dissolution of the Ag_3_PO_4_ layer in the AZ-NC heterostructure or the oxidation of KL support. Although these issues are not as severe at lower dye loads due to less stress on the AZKL catalyst, they still lead to a 20.5% decrease in efficiency after four cycles.

The photocatalytic efficiency of the AZKL nanocomposite in treating MB dye contaminant was thoroughly assessed and compared with other nanocomposites documented in existing literature, as presented in Table 3. The results showed that the AZKL nanocomposite demonstrated exceptional effectiveness in removing MB dye, achieving a greater efficiency and kinetic rate than all other Zn and Ag- based nanocomposite photocatalysts in literature (e.g., CuO/ZnO, rGO@ZnO/CuO, Fe_3_O_4_/rGO@ZnO, Ag/g-C_3_N_4_/LaFeO_3_, and Ag_3_PO_4_/WO_3_), except for Ag_3_PO_4_/GO/NCDS-2 (Table 3). The remarkable performance of the AZKL nanocomposite can be largely attributed to the combined effects of KL, which not only boosts the adsorption capacity for MB dye onto the catalytic sites but also plays a crucial role in stabilizing the AZ-NC p-n heterojunction and promoting effective charge separation. This synergy ultimately leads to a significant reduction in electron-hole recombination, thereby enhancing the overall photodegradation process. This suggests that AZKL could play a significant role in the advancement of photocatialytic technologies, offering a promising solution for environmental remediation applications, particularly in the degradation of harmful organic pollutants. To verify the chemical stability of the composite, XRD analysis was conducted before and after a series of four reuse cycles, as illustrated in Fig. 2S** (ESI)**. The XRD patterns showed no notable alterations in crystallinity or phase composition of AZKL before and after reuse cycles, indicating that the heterostructure composite of AZ-NC (Ag_3_PO_4_@ZnO) and KL support remained stable. Additionally, the lack of new peaks or shifts in existing peak positions confirmed that the composite maintained its integrity without undergoing chemical changes or degradation during operational use. To further address environmental concerns, the potential for leaching of metal species from the AZKL nanocomposite during reuse cycles was evaluated by examining the treated water solution for Zn and Ag metal ion release after a 4-cycle experiment. A digestion method was also employed to assess the change in Zn and Ag metal ions within the AZKL nanocomposite before and after the reuse cycles, followed by analysis using atomic absorption spectroscopy (AAS). The AAS analysis in Table 3S **(ESI)** revealed minimal leaching of Zn²⁺ and Ag⁺ ions from AZKL (accumulative amount of 0.089 mg/L for Zn²⁺ and 0.166 for Ag⁺ across the four cycles), indicating that the AZKL nanocomposite is environmentally safe.

**Table 3:** A comparison of the photocatalytic efficiency of the synthesized AZKL nanocomposite for removing MB dye against findings from existing literature.

Catalysts	Operational conditions	RE%	K_app_	references
CuO/ZnO	0.4 g/L catalyst and 20 ppm MB dye for 105 min (visible light)	82	0.017	^92^
rGO@ZnO/CuO	0.4 g/L catalyst and 10 ppm MB dye for 105 min (150 W tungsten halogen lamp)	90	0.02213	^93^
Fe_3_O_4_/rGO@ZnO	2 g/L catalyst and 100 ppm MB dye for 90 min (500 W tungsten halogen lamp)	98.5	0.0533	^94^
Ag_3_PO_4_/GO/NCD_S_−2	1 g/L catalyst and 10 ppm MB dye for 2.5 2.5 min (250 W xe lamp)	95	0.6051	^95^
KBiO_3_/nano-Ag_3_PO_4_	0.4 g/L catalyst and 20ppm MB dye for 240 min (300 W xe lamp)	96.7	0.0143	^96^
Ag/g-C_3_N_4_/LaFeO_3_	0.3 g/L catalyst and 10ppm MB dye for 90 min (300 W xe lamp)	98.97	0.0414	^97^
Ag_3_PO_4_/WO_3_	1 g/L catalyst and 10 ppm MB dye for 60 min (300 W xe lamp)	95	0.048	^98^
KL-Ag_3_PO_4_@ZnO (AZKL; 1:1 wt%)	4.92 catalyst, 25 ppm MB dye, and 0.03% H_2_O_2_ for 40 min (500 W visible light)	99.4	0.113	**This work**

This is further validated by measuring the effect of AZKL (relative to AZ-NC) on the inhibition of *Escherichia coli* (*E. coli*) colony-forming units (CFU) growth culture (toxicity test), as illustrated in ***Section S1.4*** **and** Table 4S **(ESI).** Specifically, using AZKL to treat the MB solution decreased the residual inhibitory effect of AZ-NC on *E. coli* growth by 17%, reducing the %inhibition effect from 43% with AZ-NC to 26% with AZKL. Furthermore, the MB solution treated with AZKL had a minimal inhibitory effect on *E. coli* growth (at only 11% inhibition) compared to the 37% inhibition observed with the AZ-NC-treated MB solution (Table 4S). The observation suggests that AZKL demonstrates minimal toxicity compared to the pure AZ-NC heterojunction. This reduced toxicity is attributed to the contribution of KL functionalities, which help to decrease ROS-induced cytotoxicity for *E. coli* cells ^78,79^. Additionally, the encapsulating properties of KL polymeric nature for AZ-NC nanocatalyst likely decrease the dissolution and photocorrosion rates of the Ag_3_PO_4_@ZnO heterostructure during photocatalysis, thereby minimizing the risk of toxic metal ion release (like Zn²⁺ and Ag⁺, Table 3S, **ESI**) into the treated water. The numerous functional groups found in KL support (e.g., phenolic hydroxyls and carboxyls) can also adsorb or chemically interact with intermediate byproducts formed during the photocatalytic degradation of MB dye, facilitating their further breakdown into non-toxic mineral forms like CO₂ and H₂O ^80^. This detoxification ability is supported by existing research that highlights lignin’s role in scavenging reactive species and stabilizing intermediate reaction products. These findings revealed that the AZKL nanocomposite serves a dual purpose: it effectively treats MB dye solutions while reducing the toxic effects of the pristine AZ-NC heterojunction catalyst on *E. coli* growth. This aligns with industrial needs for sustainable wastewater treatment, where secondary contamination risks must be minimized. According to the above findings, it can be concluded that the constructed AZKL nanocomposite adheres to green chemistry principles, ensuring that the nanocomposite effectively degrades dyes while preventing secondary pollution, thus mitigating significant environmental risks associated with the use of nanomaterials. These results underscore the robustness of the ternary AZKL nanocomposite, making it suitable for extended use in wastewater treatment.

### Realistic application of solar-driven photocatalytic treatment of textile wastewater

As the best performer, the performance of AZKL nanocomposite in removing MB dye (25 mg/L) was tested under conditions simulating high salinity (0.2 M NaCl) and varying extreme pH levels (3, 7.5, and 11) typical of industrial effluents, as illustrated in Fig. 5**(b**). The results in Fig. 5**(b)** indicated that the effectiveness of the AZKL nanocomposite was significantly influenced by both pH and salinity, especially when surpassing the limits set by RSM modeling (**Eq.** 5 and Table 1). At a near-neutral pH of 7.5, the AZKL exhibited optimal efficacy in removing MB dye, showing only a slight reduction in efficiency from 99.4 to 94.6% when shifting from non-saline to highly saline conditions. At an extremely acidic pH of 3, the AZKL maintained its dye removal performance in non-saline environments, experiencing only a minor efficiency loss of 7% compared to pH 7.5. However, its efficiency dropped significantly to 76.8% when transitioning from pH 3 to highly alkaline pH 11 in a non-slaine environment. Under saline conditions, the efficiency fell to 81.5% at pH 3 and dropped dramatically to 61.2% at pH 11 (Fig. 5**(b))**. As a fact, the AZKL nanocatalysts exhibit high stability in both nonsaline and saline environments at near-neutral pH, largely due to the role of KL in stabilizing the catalysts and enhancing charge transfer. This helps counteract the negative effects of increased Cl⁻ ion concentrations (scavenging effects) on the availability of ROS and the degradation rate of MB dye under saline conditions. Additionally, the functional groups in the KL structure improve the adsorption of MB molecules, leveraging the strong electrostatic attraction between the positively charged MB⁺ and the negatively charged surface at pH 7.5 (higher than the PZC of 4.5), which mitigates the adverse influence of Cl⁻ ions on the photocatalytic oxidation process. However, at a low acidic pH of 3, the AZ-NC nanocatalyst (Ag_3_PO_4_@ZnO) may become unstable, leading to a greater potential for leaching of Ag^+^/Zn^2+^ ions into the treated solution. This not only compromises the structural integrity of the catalyst but also disrupts the effective movement of charge carriers within the AZKL nanocomposite. The protonation of KL’s surface functionalities at this acidic pH also increases repulsion between the positively charged surface and the cationic MB dye, which reduces the effectiveness of both adsorption and photocatalysis. In extremely alkaline conditions (pH 11), the performance of the composite is most negatively impacted by high salinity due to the excessive deprotonation of KL’s functional groups and the dissociation of H_2_O_2_, while the scavenging effect of Cl⁻ further diminishes ROS generation, resulting in a significant drop in efficiency. These findings highlight the robustness of AZKL in saline conditions at near-neutral and acidic pH levels. This observation indicates that the AZKL catalyst is stable and likely suitable for practical application in the catalytic breakdown of organic dye pollutants in actual wastewater treatment, particularly at low dye concentrations.

Accordingly, the practical use of the AZKL nanocomposite for photocatalytic removal of organic pollutants in actual textile wastewater from local industry was assessed under direct sunlight, as shown in Table 4. The wastewater sample collected exhibited a nearly neutral pH (6.87), with only a slight change in pH (pH 6.59) after treatment, all staying within acceptable discharge limits. After treatment, the total dissolved solids (TDS) in the wastewater were significantly reduced from 2790.6 mg/L to 1420.1 mg/L, and salinity levels decreased from 1570 mg/L to 1163 mg/L. The TDS/salinity levels of treated effluents are within the acceptable limits set by WHO/EPA, which specifies that TDS should be less than 2000 mg/L in treated industrial effluents before discharge. The textile wastewater also experienced a notable reduction in BOD_5_ and COD levels, decreasing by 71.04% and 57.5%, respectively, after photocatalytic treatment with AZKL nanocomposite (Table 4). Notably, the COD/BOD ratio dropped from 2.62 in the untreated effluent to 1.47 post-treatment, indicating a change in the composition of organic matter and improved biodegradability. A lower COD/BOD ratio (less than 2) suggests a significant reduction of non-biodegradable components, leaving primarily biodegradable substances that are suitable for biological treatment ^81^. This reduction highlights the effectiveness of AZKL nanocomposite in breaking down organic pollutants into CO₂ and H₂O, as evidenced by the substantial decrease in the TOC value from 120 mg/L (before treatment) to 25.9 mg/L (after treatment). The UV-visible spectra of wastewater effluent, both prior to and following treatment in Fig. 5**(c**), also confirm the effective degradation and mineralization of organic pollutants through the use of AZKL nanocomposite under sunlight exposure. The results achieved comply with the discharge regulations for industrial wastewater established by entities such as the EU Water Framework Directive (WFD) and the EPA. These standards typically mandate that TOC levels remain below 100 mg/L and that the ratio of COD to BOD is under 2. This is to guarantee that residual organic materials in the effluents are more biodegradable when released into water bodies, thereby safeguarding aquatic ecosystems and enabling safe water reuse.

**Table 4:** A case study on the practical capability of AZKL nanocomposite for treating a real industrial wastewater effluent under sunlight irradiation (Conditions: 4.92 g/l catalyst dosage and 0.03% H_2_O_2_ co-catalyst for 120 min under direct sunlight with an agitation speed of 100 rpm without pH adjustment).

Water quality indicators	Before treatment	After treatment	Unit
**Total Dissolved Solids (T.D.S.)**	2790.6	1420.1	mg/L
**Salinity (as NaCl)**	1570	1163	mg/L
**pH @ 25** ^**o**^**C**	6.87	6.59	-
**TOC**	120.0	25.9	mg/L
**BOD**	95.3	27.6	mg/L
**COD**	250.0	40.5	mg/L
**COD/BOD ratio**	2.62	1.47	-

A comprehensive economic analysis was also performed to evaluate the cost-effectiveness of wastewater treatment using the AZKL nanocomposite. Based on the market price over 2024, the production cost of KL, sourced from valorizing Egyptian pulp waste, is estimated to be $0.79 ± 0.31 per kg, while the initial production cost for the AZ-NC heterojunction (Ag_3_PO_4_@ZnO) is projected at $18.02 ± 1.03 per kg. Additional costs are also involved at $0.95 ± 0.17 per kg for energy expenses (e.g., mixing, drying, and thermal treatments) and $3.01 ± 0.57 per kg for indirect costs (like skilled labor and overhead). Therefore, the total estimated production cost for 1 kg of the AZKL nanocomposite, which consists of a 1:1 ratio of KL to AZ-NC photocatalyst, is $13.76 ± 1.04, factoring in energy, labor, and raw chemical prices. The average energy consumption for each treatment cycle using visible light is estimated to be $0.015 over 1 h (0.5 kWh), based on an electricity rate of $0.03/kWh in Egypt. Consequently, treating one cubic meter of wastewater using the AZKL nanocomposite (at 4.92 kg/m³) is projected to cost $67.71 ± 2.56 to meet EU WFD and EPA standards. Assuming the potential capability of AZKL nanocomposite for 4 reuse cycles with good performance stability, the treatment cost should be thus declined to approximately $17.12 ± 0.82 per m³ per cycle when considering waste valorization, solar compatibility, and regulatory-grade pollutant removal. This estimated techno-economical cost analysis for utilization of AZKL in wastewater treatment is recognized as more effective than conventional methods, which utilize energy-intensive UV/O_3_ systems or homogeneous catalysis (like Fenton-like reaction) and generate secondary pollution during treatment.

Accordingly, the treated effluent metrics demonstrate the successful detoxification of wastewater pollutants through AZKL, which shows promise for treating textile wastewater with high photocatalytic efficiency in converting complex dye and other organic pollutant molecules into biodegradable substances, inorganic compounds, CO_2_, and H_2_O when exposed to the light source (visible or sunlight). This is validated by the identification of intermediates generated during photocatalysis using a gas chromatography-mass spectroscopy (GC-MS) technique (refer to Sect. 3.5 and **Table 5 S (ESI)**). The reduction in TDS and water salinity also indicates the effectiveness of AZKL in tackling complex organic and inorganic pollutants, confirming its suitability for industrial wastewater treatment in real-world settings. Such phenomenon is ascribed mainly to the role of KL surface functional groups that facilitate the adsorption of dissolved salts and heavy metal ions from wastewater solution during photocatalytic treatment, helping to avoid secondary contamination from heavy metals in textiles (e.g., Pb²⁺, Cd²⁺, and Cr⁶⁺) while improving the photocatalytic removal of organic contaminants under light irradiation. Future research is thus recommended to further assess and optimize the effectiveness of AZKL in treating both organic and inorganic contaminants (e.g., heavy metals and emergent organic pollutants like pharmaceutical antibiotics) within complex water mixtures that reflect actual conditions.

### Photocatalytic degradation mechanism of MB dye onto AZKL nanocomposite

Based on the outcomes of this study, a possible charge separation and transfer mechanism for the enhanced activity and stability of the KL-supported Ag_3_PO_4_@ZnO composite was proposed (Fig. 6**(a)**). The examination of UV-vis DRS and the Tauc plot shown in Fig. 3S**(a-c) (ESI)** reveals that the Ag_3_PO_4_ photocatalyst can absorb visible light with an E_g_ value of 2.34 eV, while pure ZnO absorbs UV light with an E_g_ value of 3.22 eV. According to Mulliken electronegativity theory (Eqs. 7 and 8), the band structures (i.e., the potential energy level conduction (CB) and valance (VB) bands) of the ZnO and Ag_3_PO_4_ photocatalysts were determined. The theoretical calculations revealed that the ECB potentials for Ag_3_PO_4_ and ZnO are 0.28 eV and − 0.32 eV, respectively, while their EVB potentials are 2.62 eV for Ag_3_PO_4_ and 2.90 eV for ZnO.

7 $${\rm E_{VB}=x-E_{e}+0.5E_{g}}$$

8 $${\rm E_{CB}=E_{VB}-E_{g}}$$

Where *x* is the absolute electronegativity of the semiconductors (*x* = 5.79 for ZnO and 5.95 for Ag_3_PO_4_), while E_e_ is the energy of free electrons at the hydrogen scale (~ 4.5 eV).

When ZnO and Ag₃PO₄ are hybridized in the AZ-NC structure, they create a Type-II heterojunction. When exposed to light, electrons in the EVB of both materials are excited to their respective CB, resulting in the formation of holes in the VBs. Due to the energy alignment and the favorable energy gradient (− 0.32 eV for the ZnO CB compared to 0.28 eV for the Ag₃PO₄ CB), the photogenerated electrons from the CB of ZnO move to the CB of Ag₃PO₄, while the holes from Ag₃PO₄ shift to the VB of ZnO ^82^. This charge transfer induces the formation of an internal electric field (IEF) at the interface of AZ-NC p-n heterojunction ^83^. The interaction between the KL substrate and Ag₃PO₄ NPs also leads to the partial reduction of silver ions (Ag⁺) to metallic silver (Ag⁰), as confirmed by XPS analysis of the AZKL nanocomposite (**Fig. 7S and Section S1.5 in the ESI file**). The conversion of Ag⁺ to Ag⁰ is thermodynamically advantageous because the redox potential of silver (E◦ = +0.799 V) is compatible with the electron-rich surroundings created by the phenolic hydroxyl groups in lignin ^84^. This reduction step stabilizes the silver NPs and boosts charge transfer efficiency within the ternary AZKL nanocomposite. The resulting metallic silver also acts as an electron sink, forming a Schottky junction with ZnO and Ag₃PO₄ to enhance charge separation and improve catalytic performance. In addition, the KL and its electron sink effect worked toward facilitating the charge transfer during the photocatalytic activation, diminishing the recombination phenomenon and consequently increasing the overall photocatalytic activity ^85–87^ The formed p–n heterojunctions can also enhance the separation of photogenerated charge carriers through excited electron and hole transition between ZnO and Ag_3_PO_4_, thereby improving their photocatalytic efficiency. In the process of photocatalysis, the charge carriers generated engage in redox reactions to produce ROS radicals, which are essential for the photocatalytic breakdown of MB dye. Note that the standard redox potentials for various reactions are provided, including E°(H_2_O/∙OH = 2.88 eV), E°(H_2_O_2_/∙OH = 0.94 eV), E°(HO-/∙OH = 1.9 eV), and E°(O_2_/∙O_2_− = −0.33 eV) vs. Normal Hydrogen Electrode (NHE)) ^88^. These values indicate that the VB potential of ZnO is well-suited for oxidizing HO-, H_2_O, and H_2_O_2_ to produce ∙OH radicals. These ∙OH radicals have the highest oxidation potential (E°= +2.80 V vs. NHE), making them vital in the photocatalytic degradation process ^35,85^. Conversely, the CB edge potential of Ag_3_PO_4_ is not thermodynamically favorable for reducing O_2_ to form ∙O_2_^−^ radicals compared to the CB edge of ZnO, suggesting that this reaction has a limited effect on the photocatalytic degradation process. As ea fact, the lower oxidation potential of ⋅O₂⁻ radicals (E°= −0.13 V vs. NHE) suggests that it is less reactive than ⋅OH radicals, yet it still has a minor role in oxidative degradation processes, especially as an intermediate in ROS pathways.This verified the substantial contribution of **∙**OH and hole (*h*^*+*^) in the photocatalytic degradation mechanism of organic dye onto AZKL under light irradiation (Fig. 6**(a))**.

The scavenger study was conducted to confirm the key ROS responsible for the photocatalytic degradation of MB using the AZKL nanocomposite, as illustrated in Fig. 6**(b).** Notably, the introduction of isopropanol and acetonitrile significantly decreased the efficiency of MB photodegradation, suggesting that *h*^*+*^ and •OH radicals play a major role in the photocatalytic degradation process. In contrast, the •O_2_^−^ radical formation had a minimal effect on the photocatalytic degradation of MB using the AZKL nanocomposite. Also, it was noticed that H_2_O_2_ serves as an electron acceptor, producing ⋅OH through reduction, but its contribution is relatively small (accounting for only about 10%) compared to the direct formation of ⋅OH radicals. The overall impact of ROS on the photocatalytic degradation of MB dye onto AZKL can be ranked as *h*^*+*^ species > •OH radicals > •O_2_^−^ radicals > H_2_O_2_. These findings were further validated using fluorescence photoluminescence (FPL, **Fig. 8S(a-c)**), providing further confirmation of the active species involved in the photocatalytic mechanism. In the FPL analysis, coumarin, 4-chloro-7-nitrobenzo-2-oxa-1,3-diazole (NBD-Cl), and 1,3-diphenylisobenzofuran (DPBF) were employed as fluorescent probe molecules for hydroxyl radicals (•OH), superoxide radicals (•O₂^-^), and singlet oxygen (¹O₂), respectively. As can be seen in **Fig. 8S(a) (ESI)**, the fluorescence emission peak appears at 450 nm, with its intensity progressively increasing, suggesting a rise in •OH concentration as the illumination time extends. This phenomenon occurs due to the continuous formation of •OH radicals, which subsequently react with coumarin to yield 7-hydroxycoumarin, leading to an incremental fluorescence intensity over time. Likewise, the reaction of photogenerated •O₂^-^ radicals with NBD-Cl showed a fluorescence peak centered at 550 nm (**Fig. 8S (b), ESI)**, with a gradual decline in fluorescence intensity as illumination time progresses. This observation suggests the dominant role of •OH radicals (compared to O₂^-^ radicals) in the reaction mechanism, acting as the primary active species in the photocatalytic degradation pathway of MB dye, as verified by GC-MS analysis (**Table 5 S**,** ESI**) and bandgap structure of AZKL nanocomposite photocatalyst (Fig. 6**(a)).** Furthermore, singlet oxygen (¹O₂) detection was performed using DPBF as a probe molecule, which displays a strong fluorescence signal with a peak at 475 nm (**Fig. 8S(c**)), with is gradually intensified with increasing illumination duration. These findings collectively indicate that the AZKL-HNBs catalyst is capable of generating •OH, •O₂^-^, and ¹O₂ species, where ⋅OH radical formation is the main contributor in the reaction pathway. This suggests the contribution of *h*^*+*^ active site in the direct oxidation of water, H_2_O_2_, or hydroxide ions to generate ⋅OH radicals.

Accordingly, it can be suggested that the improved degradation of MB dye on AZ-NC in the presence of H_2_O_2_ is attributed to enhanced ROS generation (including •OH and •O_2_⁻ radicals) through two processes: Ag_3_PO_4_ facilitates the reduction of H_2_O_2_ to •OH using conduction band electrons, while ZnO encourages the formation of •O_2_⁻ via hole-mediated oxidation during the charge transfer mechanism in the AZ-NC heterojunction under light irradiation. However, in the case of AZKL, the polyphenolic structure of KL support could introduce a competing reaction mechanism, reacting with H_2_O_2_ molecules to diminish its effectiveness in ROS generation (i.e., scavenge ROS through electron donation) under light irradiation ^89,90^. Despite these competing effects, the electron-donating groups on the KL support contribute to improving charge separation, resulting in limited or opposing effects ^89,91^. This demonstrates the dual functional role of KL support as both a stabilizer and a scavenger, modifying the mechanistic behavior of H_2_O_2_ in the AZKL-based photocatalytic system against MB dye (compared to the pristine AZ-NC heterojunction photocatalytic system) as supported by RSM-FCCD analysis (Fig. 4**and** Table 2). This complex interplay of factors underscores the importance of understanding both the linear and interaction effects to optimize the photocatalytic degradation process effectively.

To elucidate the photocatalytic degradation process of MB using AZKL, a GC-MS analysis was conducted to identify the main intermediates produced during the photocatalytic degradation under optimal conditions. The GC-MS results in **Table 5 S (ESI)** revealed several intermediates, such as N, N-dimethyl-p-phenylenediamine (C₈H₁₂N₂), hydroquinone, catechol, oxalic acid, and formic acid. These intermediates suggest that MB dye is gradually broken down into non-toxic end products (e.g., CO₂ and H₂O), demonstrating the efficacy of the AZKL nanocomposite in treating wastewater by completely mineralizing organic pollutants (see Sect. 3.4). The initial by-product expected to form is N, N-dimethyl-p-phenylenediamine, which appears due to the cleavage of methyl groups from the MB molecule (with m/z peaks at 120, 91, and 65). Hydroxyl radicals (⋅OH) generated can also target the aromatic rings of MB, leading to hydroxylation and the formation of phenolic compounds like hydroquinone and catechol, which share similar m/z peaks of 110, 82, and 54. Continued oxidation by ⋅OH radicals further breaks down these phenolic compounds into smaller aliphatic acids, such as oxalic acid (m/z peaks at 90 and 45), which then oxidizes to form formic acid (m/z peaks at 46 and 29), confirming a complete mineralization pathway.

## Conclusion

This study employed circular economy principles to create a novel nanocomposite photocatalyst by repurposing pulping black liquor waste to produce kraft lignin (KL) biopolymer. This KL biopolymer serves as a supportive matrix for the Ag_3_PO_4_@ZnO (AZ-NC) p-n heterojunction, enhancing structural integrity and performance stability under different water conditions. The resulting KL-based Ag_3_PO_4_@ZnO nanocomposite (AZKL) demonstrated superior performance compared to the bare AZ-NC heterojunction in removing MB dye in both dark and visible light conditions. This improvement is attributed to KL’s dual role in facilitating charge carrier separation and attracting organic dye molecules to the active sites. Response surface methodology (RSM) analysis confirmed that AZKL effectively removed a high concentration of MB dye (25 mg/L) by 98.2–99.4% at a near-neutral pH of 7.48 using a catalyst dose of 4.92 g/L and a low concentration of H₂O₂ (0.03%) within 40 min of visible light exposure with good stability over 4 reuse cycles, showcasing its potential for textile wastewater treatment. In practical wastewater treatment, the AZKL nanocomposite utilizes the photocatalytic features of ZnO and Ag₃PO₄, combined with the adsorption and stabilization capabilities of KL support, to effectively eliminate both organic and inorganic pollutants. This effectiveness is demonstrated by a notable decrease in TDS, TOC, COD, and BOD metrics by 1.96, 4.6, 6.2, and 3.5 times, respectively. This combination also improves charge separation and increases the generation of reactive oxygen species (ROS), with hydroxyl (•OH) and hole (*h*^*+*^) identified as the main components in the photocatalytic mineralization pathway of MB dye, as confirmed by the radical scavenger test and GC-MS analyses. Accordingly, the toxicity study involving *E. coli* also demonstrated that the treated effluent discharged using AZKL is safe, particularly when compared to the pristine AZ-NC nanocatalysts. In this context, KL serves as a protective layer that prevents the dissolution and photocorrosion of AZ-NC nanocatalysts during the photocatalytic process. Due to its excellent efficiency, stability, and suitability for actual wastewater treatment, the developed AZKL nanocomposite is expected to show great potential for the removal of other organic pollutants, such as dyes, pharmaceuticals, and other emerging contaminants. Upcoming research should thus aim to enhance the synthesis process on a larger scale and investigate the composite’s effectiveness in managing complicated industrial wastewater.

## Electronic supplementary material

Below is the link to the electronic supplementary material.

Supplementary Material 1

## Data Availability

The data that support the findings of this study are available from the corresponding authors upon reasonable request.
