# Supplementary material for: Ag₃PO₄@ZnO kraft lignin composite for optimized photocatalytic degradation of methylene blue using response surface methodology
Source: Sci Rep. 2025 Jun 20;15:20165. doi: 10.1038/s41598-025-05597-7 (PMC12181256; doi:10.1038/s41598-025-05597-7)
Supplement: Supplementary file 1 — Supplementary Material 1 [file 41598_2025_5597_MOESM1_ESM.docx]

**Ag₃PO₄@ZnO kraft lignin composite for optimized photocatalytic degradation of methylene blue using response surface methodology**

**Marwa S. Abdelkader^1^; Sherif A. Younis^1^; Esraa M. El-Fawal^1^****^*^; Hager R. Ali^1^; Hosny Ibrahim^2^**

***^1^****Analysis and Evaluation Department, Egyptian Petroleum Research Institute, Nasr City, Cairo 11727, Egypt; ^2^Chemistry Department, Faculty of Science. Cairo University, Egypt.*

**^*^****Corresponding author:**

E-mail address [esraaelfawal@gmail.com](mailto:esraaelfawal@gmail.com) (**Orcid No: 0000-0001-7236-1970, Esraa M. El-Fawal**).

***S1. Supplementary experimental procedures***

***S1.1. Materials***

Zinc acetate di-hydrate [Zn (CH_3_COOH)_2_·2H_2_O 99.5%] and sodium hydroxide pellets (NaOH, >99%) were purchased from Merck Co. Ltd. Alkali-lignin (97% pure), silver nitrate (AgNO_3_, ≥ 99%), Degussa TiO2 (P25 > 99.5%), ZnO nanopowder (particle size < 100 nm), methyl orange (MO: purity of 85%), methylene blue dye (MB dye > 95%), benzoquinone (> 95%), sodium carbonate (Na_2_CO_3_ > 98%), and disodium hydrogen phosphate (Na_2_HPO_4_, >99.0) were purchased from Sigma-Aldrich. Other chemicals, solvents, and reagents were of analytical grade (such as NaCl (98%), HNO_3_ (70%), HCl (36%), methanol (99.5%), ethanol (70%), isopropanol (99%), and acetone (99.5%)) and procured from a local chemical company (PioChem). Deionized water (DI) was freshly prepared at the Egyptian Petroleum Research Institute (EPRI). Kraft lignin (KL) polymer was extracted from the collected black liquor waste of Kraft sugar cane bagasse process by using the acid precipitation method, following the same procedure described in our previous work ^1^.

***S1.2. Synthesis of Kraft lignin (KL)-based ZnO@Ag_3_PO_4_ ternary composite and characterization methods***

In this procedure, the Ag_3_PO_4_@ZnO nanocomposite was initially created through an in-situ deposition process, where a layer of Ag_3_PO_4_ was applied to the surface of ZnO nanoparticles using a simple ion exchange technique. In brief, bare zinc oxide nanoparticles (ZnO NPs) were synthesized by co-precipitating zinc hydroxide (Zn(OH)_2_) from a zinc acetate solution (0.02M, 50 mL) while adding NaOH (2 M) dropwise to achieve a pH of 12, under vigorous stirring at room temperature. After 2 hours, the resulting white Zn(OH)_2_ precipitate was thoroughly washed with deionized water and ethanol, then dried at 60 °C for 12 hours, followed by calcination at 500 °C for 3 hours to produce bare ZnO NPs. In the next step, the synthesized ZnO NPs (0.49 g, 6 mmol) were ultrasonically dispersed in 200 mL of deionized water for 30 minutes. Subsequently, a dropwise addition of 22.5 mL of AgNO_3_ solution (0.02 mol/L) was made under continuous stirring for another 30 minutes. Then, 7.5 mL of Na_2_HPO_4_ solution (0.02 mol/L) was added slowly while stirring for 1 hour to facilitate the formation of an Ag_3_PO_4_ layer on the surface of the ZnO, resulting in a binary Ag_3_PO_4_@ZnO nanocomposite (designated as AZ-NC, with a molar ratio of Ag_3_PO_4_ to ZnO of 1:40). The resulting AZ-NC p-n junction structure was filtered, washed with deionized water and ethanol, and dried overnight at 60 °C.

In the next step, the synthesized AZ-NC was impregnated onto kraft lignin (KL) biopolymer as a supported medium to prepare a ternary ZnO@Ag_3_PO_4_/KL composite (labelled as AZKL) at a fixed weight ratio of “x”= 1: 1 wt.% (KL: AZ-NC). In this regard, the synthesis of AZKL composite was conducted using a simple ultrasonic-assisted wet-impregnation method. In this step, a known amount of AZ-NC photocatalyst powder was homogeneously dispersed in ethanolic solution (100 mL) under the irradiation of ultrasonic irradiation for 1 hour at room temperature (T= 25 ± 3 ^0^C). Following that, a similar weight of KL support was added to the ethanolic suspension of AZ-NC solution under ultrasonic irradiation for another 1 hour at room temperature (T= 25 ± 3 ^0^C). The slurry product was then collected by vacuum filtration and placed in an air oven for drying at 60 ⁰C overnight. The obtained AZKL composite powder was then stored in closed amber glass vials till further use. For comparison, other AZKL nanocomposites with varying weight ratios of KL: AZ-NC (1: 0.5 and 1: 0.2) were also synthesized to evaluate and optimize the adsorption-photocatalytic performance against MB dye removal.

The X-ray diffraction (XRD, Shimadzu XD-1) was carried out using a Cu-Ka radiation source (λ = 0.154 nm) at a scan range of (4-80^o^) and step size of 0.02. To recognize the surface functionalities, a Fourier transform infrared spectrometer (FTIR) (Perkin Elmer, spectrum one) was used based on the standard KBr pellets test over the wavenumber range of 400 - 4000 cm^-1^. The morphological features and elemental composition of the prepared materials were evaluated by using (i) a high-resolution transmission electron microscope (HRTEM, JEOL JEM-2100, Japan) and (ii) a scanning electron microscope (SEM: Quanta-250 FEG, FEI, Netherlands). The optical properties of the prepared materials were also compared using (i) ultraviolet-visible (UV–vis) spectroscopy (model Jasco V-570) equipped with a diffuse reflectance (DR, Shimadzu IRS-2200) and (ii) photoluminescence (PL) analysis using Spectrofluorophotometer (SHIMADZU, RF-5301). The time-resolved PL (TRPL) decay profiles of the synthesized photocatalysts were recorded using a PL spectrometer (Model HR320, JovinYvon, France). Detection was performed with an avalanche photodiode, and the data was analyzed using a time-correlated single photon counting module (TCSPC, Model SPC-130, Becker&Hickl, Germany). The textural properties of AZKL and AZ-NC photocatalysts were obtained from the N_2_ adsorption-desorption isotherms at liquid nitrogen temperature (−196 ◦C) using Quan-tachrome Nova 3200 S automates gas sorption apparatus. Prior to the measurements, all samples were perfectly degassed at 120 ◦C for six hours under vacuum pressure 1.3 × 10^−3^ Pa. The specific surface area and pore volume were computed based on the Brunauer–Emmett–Teller (BET) and Barrett-Joyner-Halenda (BJH) theories, respectively. Dynamic Light Scattering (DLS: ZetatSizer ZS, Malvern, UK) spectroscopy was used to determine the hydrodynamic size distribution of the prepared materials. The analysis of transient photocurrent (TPC) density was conducted with a CHI660C Electrochemical Workstation under 100 W visible light ON/OFF conditions. The photoelectrochemical evaluations were performed in a 0.5 M Na_2_SO_4_ electrolyte across a frequency range of 0.01 Hz to 100 kHz at open circuit potentials of 1.0 to 1.5 V relative to Ag/AgCl. The working electrode was prepared by drop-casting a thin film of the synthesized catalysts onto fluorine-doped tin oxide (FTO) glass substrates. X-ray photoelectron spectroscopy (XPS) analysis was performed using an ESCALAB 250 system (France) equipped with monochromatized Al Kα X-rays (150 W) to investigate the surface composition and elemental states. Atomic absorption spectroscopy (AAS: Perkin-Elmer Zeeman/5000) was also used to assess the leaching content of Ag and Zn metal ions from AZKL during the photocatalytic study. For this analysis, the AZKL (before and after reuse cycles) was subjected to a thermal digestion process in an acidic mixture of nitric acid (HNO₃) and hydrochloric acid (HCl) in a 3:1 ratio, followed by thermal treatment at 120°C for 2 hours. A gas chromatograph coupled with a mass spectrometer (GC-MS, Agilent 7890B GC with 5977A MSD) was used to determine the intermediates generated during photocatalytic degradation of MB dye onto AZKL under optimum conditions. The GC was equipped with a capillary column (HP-5MS, 30 m × 0.25 mm × 0.25 µm film thickness) for the separation of intermediates using electron ionization (EI) mode set at 70 eV and mass detection range of 35–500 m/z..

***S1.3. Design matrix and variable selection for RSM-FCCD***

Unlike other RSM design matrices like Box-Behnken Design (BBD) and Factorial Design, the FCCD features three levels for each factor (low, medium, and high) and places axial points at the center of the design space ^2^. This design can efficiently model the experimental process while ensuring robust predictive capabilities to explore the linear, interactive, and quadratic effects of the independent variables (e.g., X_1_, X_2_, X_3_, and X_4_) on the optimization response factor (i.e., MB dye removal rate (mg/min)). Moreover, FCCD requires fewer experimental runs, yielding enough data to fit a quadratic model, which enhances the accuracy of optimal condition predictions. It also avoids extrapolation problems present in other designs by ensuring that all experimental data stays within the practical limits of operational parameters ^3,4^. This is crucial for identifying the best conditions in photocatalysis while reducing both experimental workload and resource use.

For RSM model analysis, different statistical terms are also utilized for model validation. These statistical terms include Adequate Precision, Adjusted and Predicted R² , Lack of Fit (LOF), p-value, F-test, C.V. % (Coefficient of Variation), and PRESS (Predicted Residual Error Sum of Squares), as shown in **Table 2**. In ANOVA and RSM analyses, Adequate Precision evaluates the signal-to-noise ratio, with values >4 indicating sufficient model resolution. Adjusted R² accounts for model complexity by penalizing unnecessary terms, while Predicted R² assesses predictive accuracy on new data, with good agreement between the two suggesting robustness. The LOF test determines if the model adequately represents the data, with non-significant results indicating a good fit. The F-test compares explained variance to residuals, where a high F-value signifies model significance, supported by a low P-value (<0.05). Additionally, Std. Dev. quantifies residual variability, and C.V.% expresses this as a percentage of the mean, with lower values reflecting precision. Finally, PRESS measures predictive error through cross-validation, with smaller values indicating better model performance. Together, these metrics ensure reliable model evaluation and optimization.

During optimization, the MB dye concentration and solution pH were also selected to mimic conditions typically found in industrial wastewater. MB dye concentrations in industrial effluents can differ greatly, typically ranging from 10 to 200 mg/L. The level of organic and chemical contaminants discharged in the waste stream can also fluctuate the pH value (between pH 4 and 12), influenced by the specific dye used, the industry, and the wastewater treatment processes employed. Textile wastewater, in particular, often contains dye levels within this range before the treatment processes, which presents a notable environmental issue. After preliminary and traditional treatment steps in industrial plants, the dye concentration in treated wastewater might fall between 2- 50 mg/L ^5,6^. Accordingly, in this study, an MB concentration of 10–25 mg/L was chosen to simulate the lower to mid-range of actual dye pollution levels in moderately or poorly treated wastewater and reflect the variability in dye usage and industrial treatment efficiencies, allowing the photocatalytic system to function under conditions that reflect real-life situations. At these concentrations, the calibration curve of MB onto UV-visible spectroscopy also showed high linearity (R^2^ > 0.99, see ***Section 2.3.2***), allowing for accurate and precise assessments of degradation rates and photocatalytic performance without the need for a pre-dilution factor (a source of experimental errors). Additionally, this concentration range is being utilized in almost all relevant research studies, making it a suitable choice for testing the efficacy of photocatalysts in treating dye-polluted water ^7–9^.

Moreover, it should be noted that the study examines the effectiveness of the AZKL photocatalyst against MB dye in the presence of H_2_O_2_ as a co-catalyst across a broad pH range of 4.0 to 9.0. While conventional homogeneous H_2_O_2_-based advanced oxidation processes (AOPs) work best at strongly acidic pH levels (2.5 to 3.0) to optimize H_2_O_2_—to—⸱OH radical conversion via protonation reaction(H_2_O_2_ +H^+^→ HO_2_^+^), the study utilized a broad pH range (4 – 9) to optimize the photocatalytic oxidation rate of MB dye via the synergistic interaction between the AZKL nanocomposite (heterogeneous) and H_2_O_2_ (homogeneous) catalysis. The wider pH range used in this study aligns better with typical industrial dye wastewater, which usually has a pH 4 -12 due to various chemicals used in processing. The chosen pH range is anticipated to promote interfacial reactions within the AZKL nanocomposite, enhancing the adsorption of MB dye through electrostatic attraction. The selection of this pH range also aims to optimize the surface chemistry of the AZKL nanocomposite, thereby improving the generation and separation of e^-^/h^+^ pairs. These pairs are essential for creating ROS, such as •OH and •O₂⁻, through their interactions with H_2_O_2_ and dissolved oxygen (O_2_) molecules in water. This enhancement allows for effective dye degradation on the AZKL surface even at nearly neutral pH, reducing the need for highly acidic conditions. In contrast, maintaining a highly acidic pH is impractical for cost and operational reasons in real-world applications if the pH level for homogeneous H_2_O_2_-based AOPs is considered.

***S1.4. Toxicity assessment and practical study***

To ensure environmental safety, a toxicity analysis was conducted to assess the impact of treated MB dye solution by ternary AZKL nanocomposite (relative to binary AZ-NC heterojunction) on *Escherichia coli* (Dh5α strain) colony-forming units (CFU). In this procedure, *E. coli* culture (10⁶ CFU/mL) was incubated in the presence of AZKL and AZ-NC samples at 37°C for 24 h under UVA (5.4 J/cm²). For comparison, the *E. coli* cultures were mixed with MB dye solution samples (1:1 v/v) (before and after treatment) using AZKL and AZ-NC photocatalysts under optimum conditions, then incubated (at 37°C for 24 h). Following that, colonies are counted post-incubation to determine the toxicity level as % CFU reduction vs. control (pure *E. coli* culture), in terms of % inhibition formula as follows.

%inhibition = $(1-\frac{{CFU}_{t}}{{CFU}_{c}})\times100$

***S1.5. XPS analysis of the AZKL nanocomposite***

The XPS spectra presented in **Fig. 7S** validate the effective integration of KL with the AZ-NC p-n heterojunction, resulting in the formation of the AZKL nanocomposite, which contains the elements C 1s, N 1s, O 1s, S 2p, Zn 2p, Ag 3d, and P 2p. The core-level spectra for C 1s, N 1s, and S 2p primarily reflect the presence of KL within the composite structure. The Zn 2p spectrum (**Fig. 7S (a))** displays two distinct peaks at binding energies of 1022.3 eV and 1045.4 eV, corresponding to Zn 2p₃/₂ and Zn 2p₁/₂, respectively, which indicate the existence of Zn²⁺ ions and confirm the incorporation of zinc species into the composite ^10^. The O 1s spectrum (**Fig. 7S (b))** is deconvoluted into four peaks, representing various oxygen-containing functional groups in the composite. The peak at 531.0 eV is associated with surface hydroxyl groups (O–H), while the peak at 532.0 eV is linked to carbonyl groups (C=O) from the lignin matrix. A peak at 533.4 eV corresponds to oxygen in phosphate groups (PO₄³⁻), indicating the interaction between ZnO and Ag₃PO₄ at the AZ-NC p-n junction interface ^11,12^. The peak at 534.6 eV represents adsorbed water molecules or weakly bonded oxygen species, further emphasizing lignin's role in enhancing the composite's surface chemistry. The Ag 3d spectrum (**Fig. 7S (c))** is also deconvoluted into four peaks, with those at 368.1 eV and 374.3 eV linked to metallic silver (Ag⁰), and peaks at 368.8 eV and 375.0 eV corresponding to Ag⁺ in the Ag₃PO₄ structure ^12^. These results indicate the successful stabilization of silver species in the composite, supported by the lignin matrix, which plays a role in stabilizing both Ag⁰ and Ag₃PO₄ species. The presence of metallic silver (Ag⁰) in the XPS spectrum suggests a partial reduction of Ag⁺ from Ag₃PO₄, likely facilitated by KL, a natural polyphenolic macromolecule with reducing properties. The phenolic hydroxyl, carbonyl, and methoxyl groups in KL can donate electrons to Ag⁺, aiding in its reduction to Ag⁰, particularly during light-induced photocatalysis. This reduction not only introduces metallic silver into the composite but also enhances its photocatalytic activity. Metallic silver also acts as an electron sink, which promotes charge separation and boosts catalytic performance. Furthermore, KL helps stabilize Ag⁰ nanoparticles, preventing their aggregation and preserving the structural integrity of the composite. This synergistic interaction between KL and Ag₃PO₄ contributes to the improved photocatalytic activity of the AZKL nanocomposite for the photodegradation of MB dye. The P 2p spectrum in **Fig. 7S(d)** also displays two distinct peaks: one at 132.5 eV associated with phosphorus in phosphate groups (PO₄³⁻) and another at 133.8 eV indicating the interaction of these phosphate groups with the lignin-composite matrix ^11^. The deconvoluted C 1s spectrum (**Fig. 7S(e**)) reveals three peaks: the first at 284.6 eV, representing C–C or C–H bonds indicative of the lignin structure; the second at 286.1 eV, linked to C–O bonds from ether or hydroxyl groups; and the third at 288.4 eV, corresponding to carbonyl (C=O) or carboxylic groups, confirming the existence of oxidized carbon species ^13,14^. The N 1s spectrum (**Fig. 7S(f)**) presents two peaks, the main one at 399.8 eV related to pyridinic nitrogen likely from nitrogen functionalities introduced by kraft lignin, and another at 401.2 eV indicative of quaternary or graphitic nitrogen, suggesting higher nitrogen oxidation or bonding to aromatic structures^15^. The S 2p spectrum reveals three peaks (**Fig. 7S(g)):** The first at 163.8 eV for S 2p3/2, representing thiol or sulfide groups; the second at 164.9 eV for S 2p1/2, reflecting spin-orbit coupling; and the third at 168.2 eV for oxidized sulfur species like sulfate (SO₄²⁻) ^16^. These results affirm the successful incorporation of kraft lignin into the AZKL nanocomposite and highlight the presence of functional groups that enhance its photocatalytic properties.

**S2. References**

1. Abdel-Aziz, M. A., Younis, S. A., Moustafa, Y. M. & Khalil, M. M. H. Synthesis of recyclable carbon/lignin biocomposite sorbent for in-situ uptake of BTX contaminants from wastewater. *J. Environ. Manage.* **233**, 459–470 (2019).

2. Nodehi, R. N., Sheikhi, R. & Gholami, M. Application of response surface methodology in the photocatalytic removal of pollutants–a systematic review: The characteristics, common mistakes, and the qualitative evaluation of articles. *Results Chem.* 101584 (2024).

3. Kashefi, S., Borghei, S. M. & Mahmoodi, N. M. Application of face-centered central composite design (fcccd) in optimization of enzymatic decolorization of two azo dyes: a modeling vs. empirical comparison. *Prog. Color. Color. Coatings* **12**, 179–190 (2019).

4. Chelladurai, S. J. S. *et al.* Optimization of process parameters using response surface methodology: A review. *Mater. Today Proc.* **37**, 1301–1304 (2021).

5. Azhar, F. H. *et al.* Methylene blue dye wastewater treatment based on tertiary stage of industrial wastewater treatment process: a review. *Emerg. Adv. Integr. Technol.* **3**, 37–51 (2022).

6. Younis, S. A., Elshafie, M. & Moustafa, Y. M. Textile effluent treatment and recycling. in *Sustainable Innovations in the Textile Industry* 505–539 (Elsevier, 2024).

7. Alzura, S. P. *et al.* Synthesis and characterization of zinc oxide nanoparticles-carbon composite derived from pineapple peel wastes for adsorption of methylene blue from solution and photocatalytic activity. *Case Stud. Chem. Environ. Eng.* 101113 (2025).

8. Dariani, R. S., Esmaeili, A., Mortezaali, A. & Dehghanpour, S. Photocatalytic reaction and degradation of methylene blue on TiO2 nano-sized particles. *Optik (Stuttg).* **127**, 7143–7154 (2016).

9. Farouq, R. Coupling adsorption-photocatalytic degradation of methylene blue and maxilon red. *J. Fluoresc.* **32**, 1381–1388 (2022).

10. Yasin, A. S., Kim, D. hyun & Lee, K. One-pot synthesis of activated carbon decorated with ZnO nanoparticles for capacitive deionization application. *J. Alloys Compd.* **870**, 159422 (2021).

11. Xu, H. *et al.* CNT/Ag3PO4 composites with highly enhanced visible light photocatalytic activity and stability. *Chem. Eng. J.* **241**, 35–42 (2014).

12. Wu, S. *et al.* Hydrothermal synthesis and visible light photocatalytic activity enhancement of BiPO4/Ag3PO4 composites for degradation of typical dyes. *Ceram. Int.* **40**, 14613–14620 (2014).

13. Talukdar, K. *et al.* Novel Z-scheme Ag3PO4/Fe3O4-activated biochar photocatalyst with enhanced visible-light catalytic performance toward degradation of bisphenol A. *J. Hazard. Mater.* **398**, 123025 (2020).

14. Zhang, J. *et al.* Facile and green synthesis of novel porous g-C3N4/Ag3PO4 composite with enhanced visible light photocatalysis. *Ceram. Int.* **43**, 1522–1529 (2017).

15. Xu, H. *et al.* g-C3N4/Ag3PO4 composites with synergistic effect for increased photocatalytic activity under the visible light irradiation. *Mater. Sci. Semicond. Process.* **39**, 726–734 (2015).

16. Abdelkader, M. S., Younis, S. A., El-Fawal, E. M., Ali, H. R. & Ibrahim, H. Hybridizing Black Liquor-Derived Kraft Lignin with Ag3po4@ Zno to Boost Tetracycline and Dye Removal Through Synergistic Adsorption-Photocatalytic Pathways. *Available SSRN 5005506*.

**Table 1S**: Performance comparison of AZKL and AZ-NC for MB dye removal as a function of agitation speed and benchmark photocatalysts under optimum operating conditions obtained by RSM analysis.

| Catalyst/agitation speed | 100 rpm | 150 rpm | 200 rpm | 250 rpm |
| --- | --- | --- | --- | --- |
| AZKL | 99.4 | 98.2 | 96.1 | 95.2 |
| AZ-NC | 84.9 | 88.6 | 90.4 | 78.5 |
| TiO2-P25* | 81.3 | - | - | - |
| ZnO (commercial)* | 68.7 | - | - | - |

*The study evaluated the performance of TiO2-P25 and ZnO as benchmark photocatalysts under the optimal conditions identified for the AZKL nanocomposite, which were determined through RSM-FCCD analysis (e.g., catalyst mass = 4.92 g/L, pH of 7.48, 0.03 % H_2_O_2_, and 100 rpm agitation speed).

**Table 2S**: Performance analysis of AZKL for MB vs. MO dye removal in dark/light conditions.

| Parameters | MB dye | MO dye |
| --- | --- | --- |
| Adsorption (%) | 54.0 | 28.7 |
| Photocatalytic (%) | 99.4 | 68.7 |
| Adsorption rate (k, min^-1^) | 0.0137 (R^2^= 0.978) | 0.006 (R^2^= 0.973) |
| Photocatalytic rate (K_app_, min^-1^) | 0.113 (R^2^= 0.929) | 0.0219 (R^2^= 0.95) |

*The study evaluated the performance of AZKL nanocomposite under optimum operating conditions obtained by RSM-FCCD analysis (e.g., catalyst mass = 4.92 g/L, pH of 7.48, 0.03 % H_2_O_2_, and 100 rpm agitation speed). Also, MO dye was detected by UV-vis spectroscopy at a maximum absorption wavelength of approximately 463-465 nm. The adsorption rate was calaculated using pseudo-first order model equation.

**Table 3S**: The AAS analysis for Zn and Ag metal ions leached from AZKL nanocomposites over four reuse cycles against MB dye removal under optimum conditions obtained by RSM-FCCD modeling.

| Cycles | Ag (mg/L) | Zn (mg/L) |
| --- | --- | --- |
| **1** | 0.013 | N.D. |
| **2** | 0.024 | 0.011 |
| **3** | 0.045 | 0.032 |
| **4** | 0.084 | 0.046 |
| **Accumulative leached amount (mg/L)** | 0.166 | 0.089 |
| **AZKL (after 4-reuse cycle)** | 0.172 | 0.109 |

**Table 4S**: CFU Reduction (%inhibition) in E. coli culture exposed to AZKL (vs. AZ-NC) catalysts along with their mixture after MB dye solution treatment under operating optimum conditions

| Sample | CFU/mL (control) | CFU/ml (treated) | CFU (% Inhibition) | Toxicity Category | |
| --- | --- | --- | --- | --- | --- |
| AZ-NC treated MB dye solution | 1.00E+06 | 6.30E+05 | 37 | Moderat-to Slightly Toxic | |
| AZKL-Treated MB dye solution | 1.00E+06 | 8.90E+05 | 11 | Non-Toxic | |
| AZ-NC eluent | 1.00E+06 | 5.70E+05 | 43 | Moderately Toxic | |
| AZKL eluent | 1.00E+06 | 7.90E+05 | 21 | Slightly Toxic | |
| * The data is shown as average values from three runs, with a standard deviation of± 3 to 4%. | | | | |  |

**Table 5S**: GC-MS identified intermediates generated during the photocatalytic degradation of MB dye onto AZKL under operating optimum conditions.

| Retention Time (min) | Compound Identified | Molecular Formula | m/z Fragments | Photocatalytic degradation pathway |
| --- | --- | --- | --- | --- |
| 1.9 | MB dye | C₁₆H₁₈ClN₃S | 284, 256, 227, 199 | Dye contaminant |
| 5.2 | N,N-Dimethyl-p-phenylenediamine | C₈H₁₂N₂ | 120, 91, 65 | Aromatic intermediate from demethylation |
| 8.7 | Hydroquinone | C₆H₆O₂ | 110, 82, 54 | Phenolic compound from hydroxylation |
| 11.4 | Catechol | C₆H₆O₂ | 110, 82, 54 | Phenolic compound from hydroxylation |
| 13.8 | Oxalic Acid | C₂H₂O₄ | 90, 45 | Aliphatic acid from ring-opening |
| 16.5 | Formic Acid | CH₂O₂ | 46, 29 | Mineralization step |


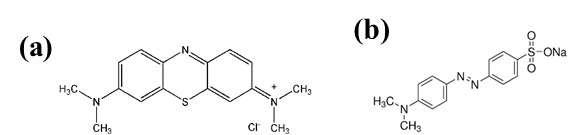


**Fig. 1S**: The molecular structure of tested dyes: (a) methylene blue (MB) dye and (b) methyl orange (MO) dye.

**Fig.2S:** The XRD patterns of AZKL, ZnO, and AZ-NC photocatalysts.

**Fig. 3S:** The optical features of the prepared AZKL, AZ-NC, and ZnO vs. Ag_3_PO_4_ photocatalysts: (a) UV-Vis DRS spectra (AZKL, AZ-NC, and ZnO) vs (b) UV-Vis DRS and Tauc plot of Ag_3_PO_4_, along with (c) Tauc plot and (d) PL emission spectra for AZKL, AZ-NC, and ZnO catalysts.


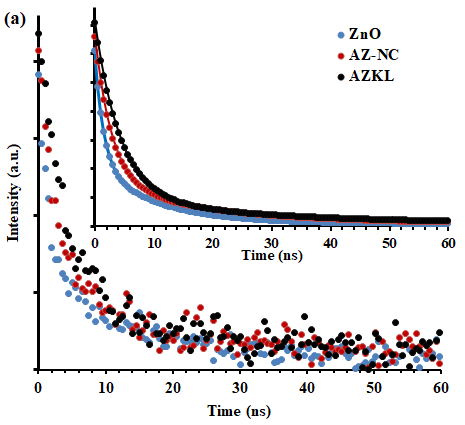


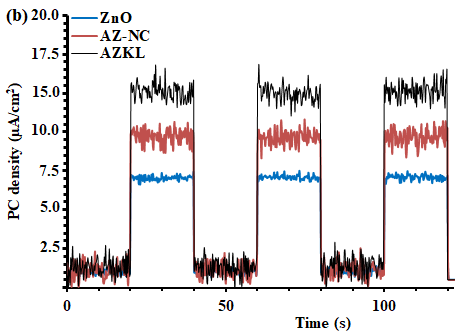


**Fig. 4S:** The photo-electro-chemical features of AZKL, AZ-NC, and ZnO photocatalysts: (a) TR-PL spectra and (b) Transient photocurrent (TPC) density profiles under visible light ON/OFF conditions.


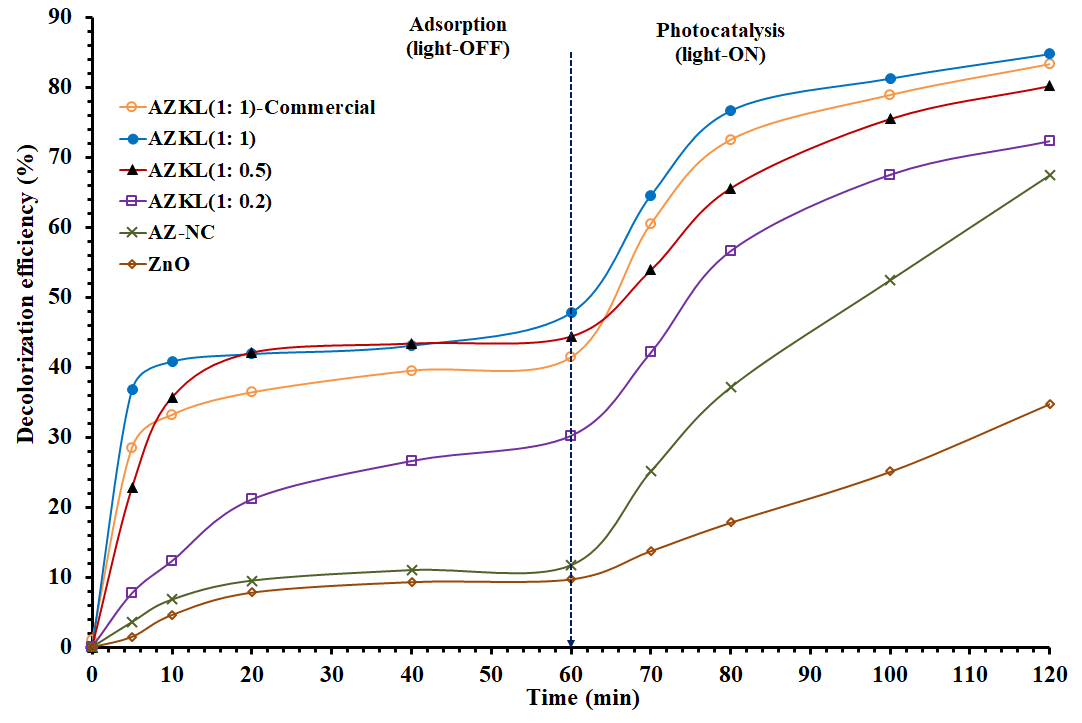


**Fig. 5S**: Adsorption-photocatalysis synergy of AZKL (at varying AZ-NC: KL weight ratios) on the removal efficiency of MB dye under Visible light-OFF/ON conditions (Operation: 10 mg/L MB dye, pH 6.5±0.5, catalyst does 2.5 g/L, agitation speed= 100 rpm, and H_2_O_2_ = 0% at room temperature of 25±3ºC).


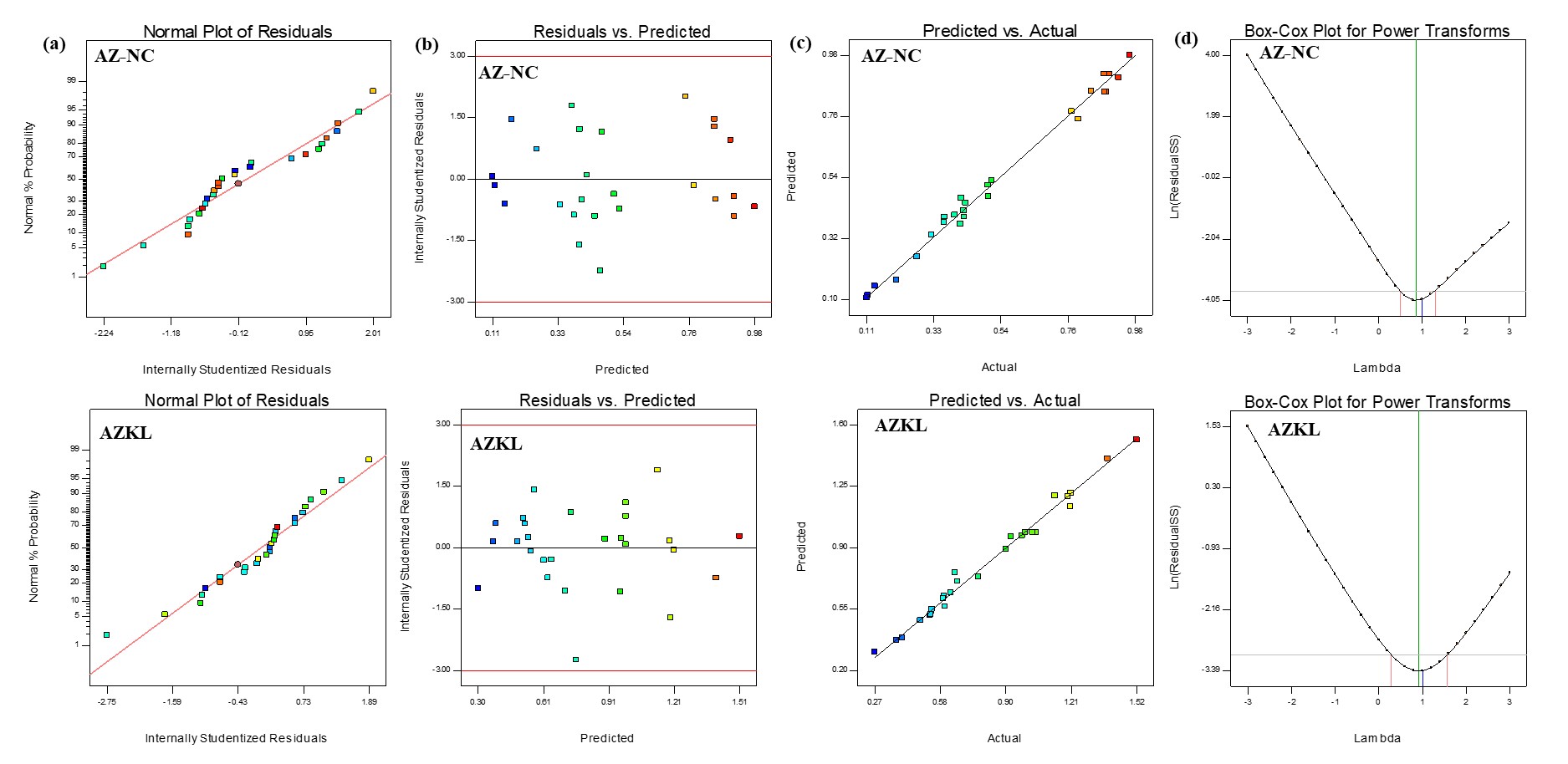


**Fig. 6S:** The fitting plots of polynomial model equations (Eqs. 5 &6) simulated for predicting MB degradation rates onto AZ-NC and AZKL photocatalysts under visible irradiation: (a) normalized residual plots, (b) residuals versus predicted values plots, (c)  experimental data versus predicted value plot s, and (d) Box-Cox plots for the models


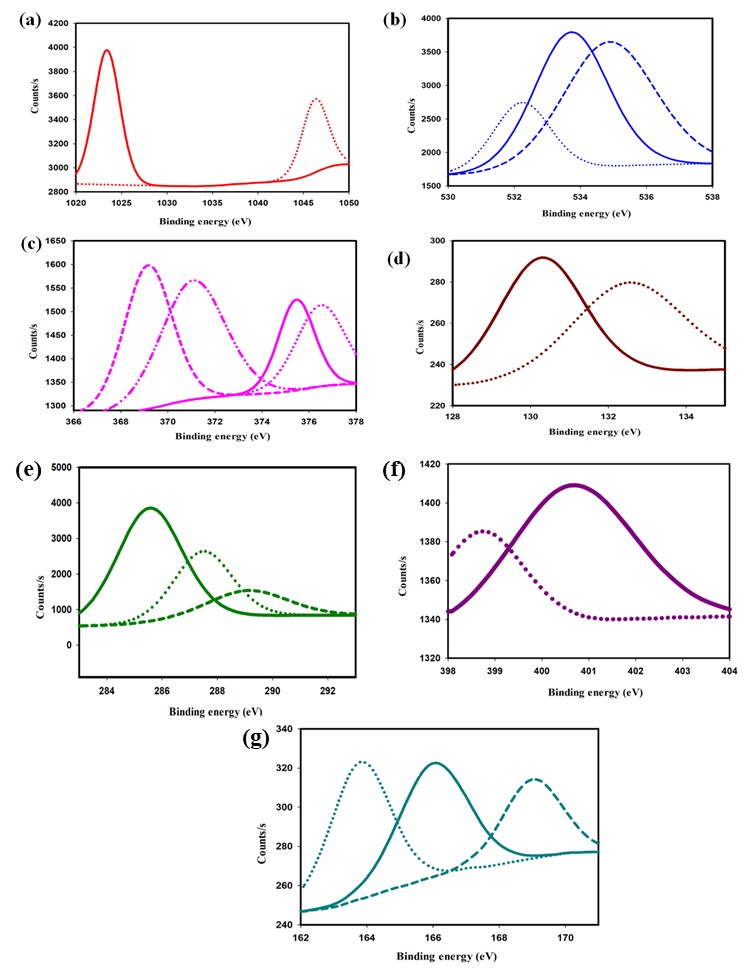


**Fig. 7S:** XPS analysis of AZKL nanocomposite: (a) Zn 2p, (b) O 1s, (c) Ag 3d, (d) P 2p, (e) C 1s, (f) N 1s, and (g) S 2p


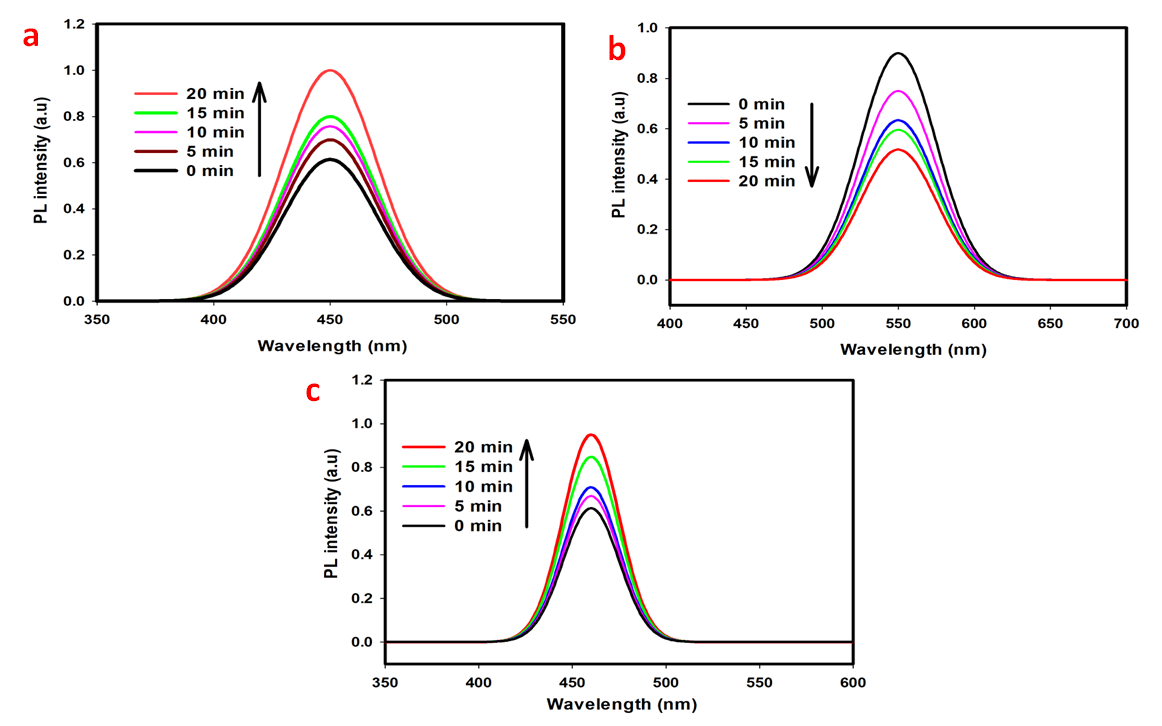


**Fig. 8S:** Fluorescent PL spectra for the active ROS species with AZKL photocatalyst: (a) •OH, (b) •O₂⁻ , and (c) ¹O₂ radicals.
